# Effect of Auricular Acupoint Stimulation on Weight in Adults with Overweight or Obesity: A Systematic Review and Meta-Analysis

**DOI:** 10.3390/healthcare14193336

**Published:** 2026-10-06

**Authors:** Mi Young Kim, Mi-Kyoung Cho

**Affiliations:** 1 College of Nursing, Hanyang University, 222 Wangsimni-ro, Seongdong-gu, Seoul 04763, Republic of Korea; miyoung0@hanyang.ac.kr; 2 Department of Nursing Science and Research Institute of Nursing Science, College of Nursing, Chungbuk National University, 1 Chungdae-ro, Seowon-gu, Cheongju 28644, Republic of Korea

**Keywords:** auricular acupressure, ear acupuncture, auriculotherapy, weight loss, systematic review, meta-analysis

## Abstract

**Objectives:** To systematically evaluate the effectiveness of auricular acupoint stimulation for weight reduction and metabolic outcomes in adults with overweight or obesity. **Design:** Systematic review and meta-analysis of randomized controlled trials. **Methods:** Six electronic databases (PubMed, Cochrane Library, Embase, CINAHL, Web of Science, and Scopus) were searched from inception to 5 February 2026. The primary outcome was body weight; secondary outcomes included body mass index, waist circumference, and metabolic markers. Methodological quality was assessed using the Joanna Briggs Institute checklist. For the primary outcome, a multilevel random-effects model was fitted using restricted maximum likelihood estimation, with small-sample-adjusted cluster-robust variance estimation (CR2) to account for dependent comparisons within studies. Subgroup analyses, meta-regression, sensitivity analyses, and publication bias assessments were also conducted. **Results:** A total of 20 studies were included in the systematic review, of which 14 contributed 15 comparisons to the primary meta-analysis of post-intervention body weight. The pooled effect was Hedges’ g = −0.21 (95% CI: −0.42 to −0.01, *p* = 0.043). Heterogeneity estimates were I^2^ = 46.6% (95% CI: 8.1–76.8%) and τ^2^ = 0.06 (95% CI: 0.01–0.21), and the 95% prediction interval ranged from −0.71 to 0.29. Formal subgroup tests and univariate meta-regression yielded *p* values below 0.05 for publication year, funding status, and quality appraisal score. Sensitivity analyses produced estimates in the same direction, although the confidence interval included the null value in some leave-one-out analyses. **Conclusions:** The pooled estimate suggested a small reduction in post-intervention body weight with auricular acupoint stimulation, but the prediction interval included the null value, indicating uncertainty in the effect across comparable settings. Further well-designed trials with standardized intervention protocols are needed to clarify the magnitude and consistency of the effect.

## 1. Introduction

Obesity—one of the most pressing global health challenges of the 21st century—continues to prevail across all age groups and geographic regions. Recent projections indicate that by 2035, the global burden of disease attributable to high body mass index (BMI) will substantially increase, with obesity-related deaths expected to rise by over 100% from 2019 levels [1,2]. Analysis of data from 204 countries and territories reveals that obesity now contributes to approximately five million annual deaths, with disability-adjusted life years exceeding 160 million globally. Projections from the Non-Communicable Disease Risk Factor Collaboration database suggest that adult overweight and obesity rates will continue their upward trajectory through 2040, with significant disparities between high-income and low-to-middle-income countries [3]. Among young adults aged below 40 years, obesity demonstrates the highest age-standardized disability-adjusted life year rates among all metabolic diseases [2].

Obesity is a major risk factor for type 2 diabetes mellitus and contributes substantially to the growing global burden of metabolic disease. Among young adults, obesity-related mortality is projected to increase considerably by 2050 [2,4]. Metabolic syndrome—encompassing dyslipidemia, hypertension, insulin resistance, and chronic inflammation—collectively contributes to increased cardiovascular morbidity and mortality. Obesity imposes substantial economic burdens on healthcare systems worldwide, with a disproportionate impact on countries with a low sociodemographic index [2].

Despite advances in obesity management, current treatment approaches face significant challenges. Conventional interventions typically yield modest long-term results, with high rates of weight regain. Pharmacological treatment may also be constrained in some patients by adverse effects, treatment burden, comorbidities, and concomitant medication use, while bariatric surgery carries surgical risks and is suitable only for select populations. These limitations have prompted growing interest in complementary and alternative medicine approaches [5], which may provide additional non-pharmacological options within comprehensive obesity management.

Among complementary and alternative medicine modalities, acupuncture has garnered substantial research attention for obesity management. Recent systematic reviews and meta-analyses demonstrate that acupuncture, when used as an adjunct to lifestyle interventions, produces statistically significant reductions in body weight, BMI, and waist circumference [6,7,8]. Network meta-analyses suggest varying efficacy profiles across acupuncture modalities, with electroacupuncture and pharmacoacupuncture showing particular promise [7,9].

Auricular acupoint stimulation represents a specific technique within the broader acupuncture family that offers unique practical advantages. Based on traditional Chinese medicine (TCM) principles, auricular acupoint stimulation involves stimulating specific ear points corresponding to body organs and systems regulating appetite, metabolism, and endocrine function. Proposed mechanisms include modulation of vagal activity and appetite-related hormonal pathways; however, these mechanisms were not directly evaluated in most included trials [10]. Compared with body acupuncture, auricular techniques may offer practical advantages such as ease of administration and the possibility of sustained stimulation through retained seeds or pellets [10].

Several systematic reviews and meta-analyses have examined auricular therapies for obesity management. Yeh et al. meta-analyzed 13 randomized controlled trials (RCTs) of auricular acupoint stimulation and found significant reductions in weight and BMI but noted high heterogeneity (I^2^ up to 88%) [11]. Previous systematic reviews have reported reductions in body weight and BMI following auricular or acupuncture-based interventions, although findings have been inconsistent and substantial heterogeneity has been reported [10,11,12,13,14,15,16]. These reviews also identified methodological limitations, including small sample sizes, variable study quality, and heterogeneous intervention protocols.

Critically, previous reviews often combined different auricular modalities, including acupressure, acupuncture, and electroacupuncture, within single meta-analyses and primarily focused on anthropometric outcomes. Consequently, the effects specifically attributable to auricular acupoint stimulation and its effects on metabolic biomarkers beyond weight and BMI remain unclear.

Understanding the mechanisms by which auricular acupoint stimulation may affect obesity is essential for clinical application and future research. In an animal model of diet-induced obesity, electroacupuncture was reported to improve leptin resistance, potentially through activation of the AMP-activated protein kinase signaling pathway [17]. A recent systematic review demonstrates that auricular stimulation significantly reduces leptin, insulin, and HOMA-IR, with vagal sensory nerve stimulation representing a key mechanistic pathway [10].

Within traditional Chinese medicine, auricular stimulation has been applied as one of several approaches to weight management. However, traditional theoretical explanations and mechanisms derived from herbal medicine should not be assumed to apply directly to auricular acupoint stimulation.

Despite growing interest and accumulating evidence, significant gaps remain in the literature. Previous systematic reviews have combined auricular acupoint stimulation with other auricular techniques (e.g., acupuncture and electroacupuncture), making it difficult to determine the effects specifically attributable to auricular acupoint stimulation. In addition, evidence regarding clinically relevant metabolic outcomes beyond weight loss remains limited.

Therefore, this systematic review and meta-analysis aimed to evaluate the effects of auricular acupoint stimulation on body weight and metabolic outcomes in adults aged 19 years or older with overweight or obesity. We also explored potential sources of heterogeneity through subgroup analyses and meta-regression.

## 2. Materials and Methods

### 2.1. Eligibility Criteria

Studies were considered eligible if they met the predefined criteria based on the PICO-SD framework, and the selection process was reported in line with the PRISMA 2020 statement [18]. The inclusion of specific study designs was justified to ensure the methodological rigor required for evidence synthesis in healthcare, consistent with guidance from the Cochrane Handbook version 6.5 for Systematic Reviews of Interventions [19]. Eligible participants (P) were adults aged 19 years or older who were classified as having overweight or obesity according to the criteria applied in the original studies. Studies involving participants younger than 19 years were excluded. The intervention (I) was defined as stimulation applied specifically to recognized auricular acupoints for the purpose of obesity or weight management. Eligible modalities included (1) auricular acupressure using seeds, beads, or pellets; (2) auricular acupuncture involving needle insertion at auricular acupoints, including intradermal or press needles; and (3) electrical stimulation applied to auricular acupoints. Interventions were categorized according to the primary mode of stimulation as acupressure, acupuncture, or electrical stimulation. Co-interventions, including dietary, exercise, lifestyle, or other adjunctive components, were not used as exclusion criteria when auricular acupoint stimulation was included as part of the intervention regimen; the presence and type of these components were recorded during data extraction. Eligible comparators (C) included no intervention, sham treatment, or other interventions used as comparison conditions, consistent with the registered review protocol. Studies without a comparison group were excluded. The primary outcome (O) was body weight. Secondary outcomes included BMI, waist-to-hip ratio, body fat, waist circumference, hip circumference, systolic blood pressure, diastolic blood pressure, leptin, fasting blood glucose, total cholesterol, and triglycerides. When multiple post-intervention time points were reported, the earliest assessment conducted at the predefined endpoint of the intervention was selected for meta-analysis. To ensure accurate estimation of pooled effect sizes, only studies providing sufficient quantitative data (sample size, mean, and standard deviation) were included in the statistical synthesis. The eligible study design (SD) was restricted to RCTs. Studies published in English or Korean with accessible full texts were eligible.

### 2.2. Information Sources

A comprehensive literature search was undertaken across six electronic databases: PubMed, the Cochrane Library, Embase (Ovid), CINAHL, Web of Science, and Scopus. Eligible publications were searched from database inception through 5 February 2026, without a gap in the publication-date coverage. The search strategy was initially developed in collaboration with a medical librarian and subsequently refined with input from a meta-analysis methodologist. Two reviewers (Cho and Kim) independently executed the finalized searches. The review protocol was prospectively registered in PROSPERO (registration number CRD42024495920) on 2 March 2024. The searches themselves were carried out in two periods: 21 December 2023–21 March 2024, for the initial search and 10 December 2025–5 February 2026, for the updated search. The latter updated the earlier search through 5 February 2026; thus, studies published between the two search periods remained within the eligible publication period.

### 2.3. Search Strategy

The search strategy was structured according to the PICO-SD framework to ensure comprehensive identification of relevant studies. Controlled vocabulary terms (e.g., MeSH in PubMed and Emtree in Embase) were combined with free-text keywords to enhance sensitivity. Search terms were organized around four main concepts: (1) auricular acupoint-related interventions, (2) overweight and obesity, (3) body weight and related outcomes, and (4) RCT methodology. Boolean operators (“AND,” “OR”) were used to combine search terms within and across concepts. Truncation and field tags were applied as appropriate for each database. Database-specific adaptations of the search syntax were implemented to account for differences in indexing systems. No restrictions on publication year were imposed. Language limits were applied to include studies published in English or Korean only. The complete electronic search strategies for each database, including detailed search strings and indexing terms, are provided in Supplementary Table S1.

### 2.4. Selection Process

All retrieved records were exported to Microsoft Excel (Microsoft Corporation, Redmond, WA, USA) for management and screening. Duplicate records were identified using a two-step procedure. First, automated sorting and filtering functions were applied to bibliographic fields—such as database source, author names, year of publication, article title, and journal name—to detect identical entries. Second, potentially overlapping records with minor discrepancies (e.g., variations in indexing format or punctuation) were manually reviewed to ensure accurate removal. Confirmed duplicates were excluded before the screening process. Following de-duplication, the two reviewers independently screened the titles and abstracts of all identified records against the predefined inclusion and exclusion criteria. Each reviewer documented decisions and reasons for exclusion during the screening phase. Articles deemed potentially eligible were retrieved in full text for further assessment.

### 2.5. Data Collection Process

Data were extracted independently by two reviewers using a predefined coding framework developed in Microsoft Excel. Prior to formal extraction, the data collection form was pilot-tested and refined to ensure clarity and consistency. For each included study, the following information was recorded: author(s), year of publication, ethical approval (institutional review board) status, funding support, sample characteristics (total sample size, number of participants in intervention and control groups, and baseline characteristics such as BMI), study design, and detailed intervention characteristics. Intervention-related variables included type of auricular stimulation, intervention tools used, number and location of auricular acupoints, intervention duration, intervention session, intervention time per session, timing of post-intervention outcome assessment, and reported outcome measures. Quality appraisal scores were also documented. After independent coding, the extracted datasets were compared to assess consistency. Any discrepancies were resolved through re-examination of the original articles and discussion between the reviewers until agreement was achieved. Subsequently, the finalized data were entered into the coding database for further analysis.

### 2.6. Data Items

Data items were predefined based on the study objectives and the PICO-SD framework. Extracted variables were categorized into study characteristics, participant characteristics, intervention details, and outcome measures. Study characteristics included first author, year of publication, country of study, ethical approval status, funding source, and study design. Participant characteristics comprised the total sample size and the number of participants allocated to intervention and control groups, where available. Intervention characteristics included type of auricular acupoint stimulation (e.g., acupressure, acupuncture, electrical stimulation), intervention tools used, number and anatomical locations of auricular acupoints, intervention duration, intervention session, intervention time per session, and timing of post-intervention outcome assessment. The primary outcome was body weight. Secondary outcomes included BMI, waist-to-hip ratio, body fat, waist circumference, hip circumference, systolic blood pressure, diastolic blood pressure, leptin, fasting blood glucose, total cholesterol, and triglycerides. For quantitative synthesis, continuous outcomes were extracted as means and standard deviations for intervention and control groups at baseline and post-intervention time points. When multiple post-intervention assessments were reported, the first measurement obtained at the predefined endpoint of the intervention was selected for analysis to ensure consistency across studies.

### 2.7. Risk of Bias Assessment

The methodological quality of included studies was appraised using the Joanna Briggs Institute Revised Checklist for Randomized Controlled Trials [20,21]. The checklist comprises 13 items, including 10 items assessing internal validity—covering bias related to selection and allocation, intervention administration, outcome measurement/detection, and participant retention—and three items addressing statistical conclusion validity. Quality appraisal was conducted independently by two reviewers, and discrepancies were resolved through discussion and re-checking of the full text until consensus was reached. Each item was operationalized for scoring as follows: responses of “Yes” (criterion clearly met) were assigned 1 point, whereas “No,” “Unclear,” and “Not applicable” were assigned 0 points. Total scores were calculated by summing item-level points, with higher scores indicating better methodological quality. Total scores were used descriptively and were not interpreted as an overall JBI risk-of-bias classification. The mean-based categorization of total scores used in exploratory subgroup analyses was a data-derived analytic variable rather than a validated risk-of-bias threshold.

### 2.8. Effect Measures

For quantitative synthesis, standardized mean differences were calculated using Hedges’ g to account for potential small-sample bias and enable comparison across studies reporting outcomes in different units [22]. For the primary analysis of body weight, effect sizes were calculated from post-intervention means and standard deviations of the intervention and control groups. Change-from-baseline body-weight scores were not combined with post-intervention values; when sufficient change-score data were available, they were synthesized separately as a secondary outcome [19]. Pooled effect estimates were presented with 95% confidence intervals (CIs). A random-effects model was applied, and between-study variance was estimated using the restricted maximum likelihood (REML) method [19]. The direction of effect sizes was defined a priori: positive values indicated an increase in the outcome variable, whereas negative values indicated a reduction in the auricular acupoint stimulation group relative to the control group.

### 2.9. Synthesis Methods

All meta-analyses were conducted using R version 4.5.1 (R Foundation for Statistical Computing, Vienna, Austria). Pooled standardized mean differences were estimated as Hedges’ g with 95% confidence intervals (CIs). For the primary meta-analysis, a multilevel random-effects model was fitted using restricted maximum likelihood (REML) estimation. To account for statistical dependence arising from multiple comparisons within the same study, effect estimates were clustered at the study level, and inference was based on small-sample-adjusted cluster-robust variance estimation (CR2) [23,24]. For conventional comparison-level analyses, including subgroup-specific pooled estimates, the sample size of a shared control group was divided across the corresponding comparisons, while the reported means and standard deviations were left unchanged [19]. Formal differences between subgroups were evaluated using omnibus moderator tests (QM) from multilevel random-effects meta-regression models. Statistical heterogeneity was evaluated using Cochran’s Q statistic, Higgins’ I^2^ statistic with its 95% confidence interval, and the estimated between-study variance (τ^2^) with its 95% confidence interval [25]. For the primary outcome, these heterogeneity measures were derived from the multilevel random-effects model, and a 95% prediction interval was additionally calculated to indicate the range of effects expected across comparable settings. Sensitivity analyses were performed using three approaches. First, leave-one-out analysis was conducted by sequentially omitting each study and recalculating the pooled estimate. Second, studies identified as outliers on the Galbraith plot were excluded, and the pooled estimate was re-estimated. Third, the primary result was compared with estimates obtained using alternative meta-analytic specifications, including random-effects models with REML and DerSimonian–Laird estimators and a fixed-effect model. For the primary outcome, subgroup analyses and meta-regression were performed to explore potential sources of heterogeneity. Formal tests of subgroup differences and univariate meta-regression analyses were based on model-based inference from multilevel random-effects models. Moderators were selected to represent clinically and methodologically relevant characteristics available across the included studies, including study characteristics, intervention features, comparator conditions, outcome assessment, and methodological quality. Given the limited number of studies and the number of moderators examined, subgroup and meta-regression analyses were considered exploratory. Despite variation in participant characteristics and recruitment settings, the included studies addressed the same prespecified clinical question and evaluated auricular acupoint stimulation for body-weight outcomes in adults with overweight or obesity. Accordingly, the populations were considered sufficiently comparable for estimation of an average intervention effect under a random-effects framework, while allowing for between-study variation in effect magnitude.

### 2.10. Reporting Bias Assessment

Potential reporting bias and small-study effects were examined by visual inspection of funnel plots and formally tested using Egger’s regression asymmetry test [26]. The trim-and-fill method was additionally applied as a sensitivity assessment of potential missing studies [27].

## 3. Results

### 3.1. Study Selection

**Figure 1:**
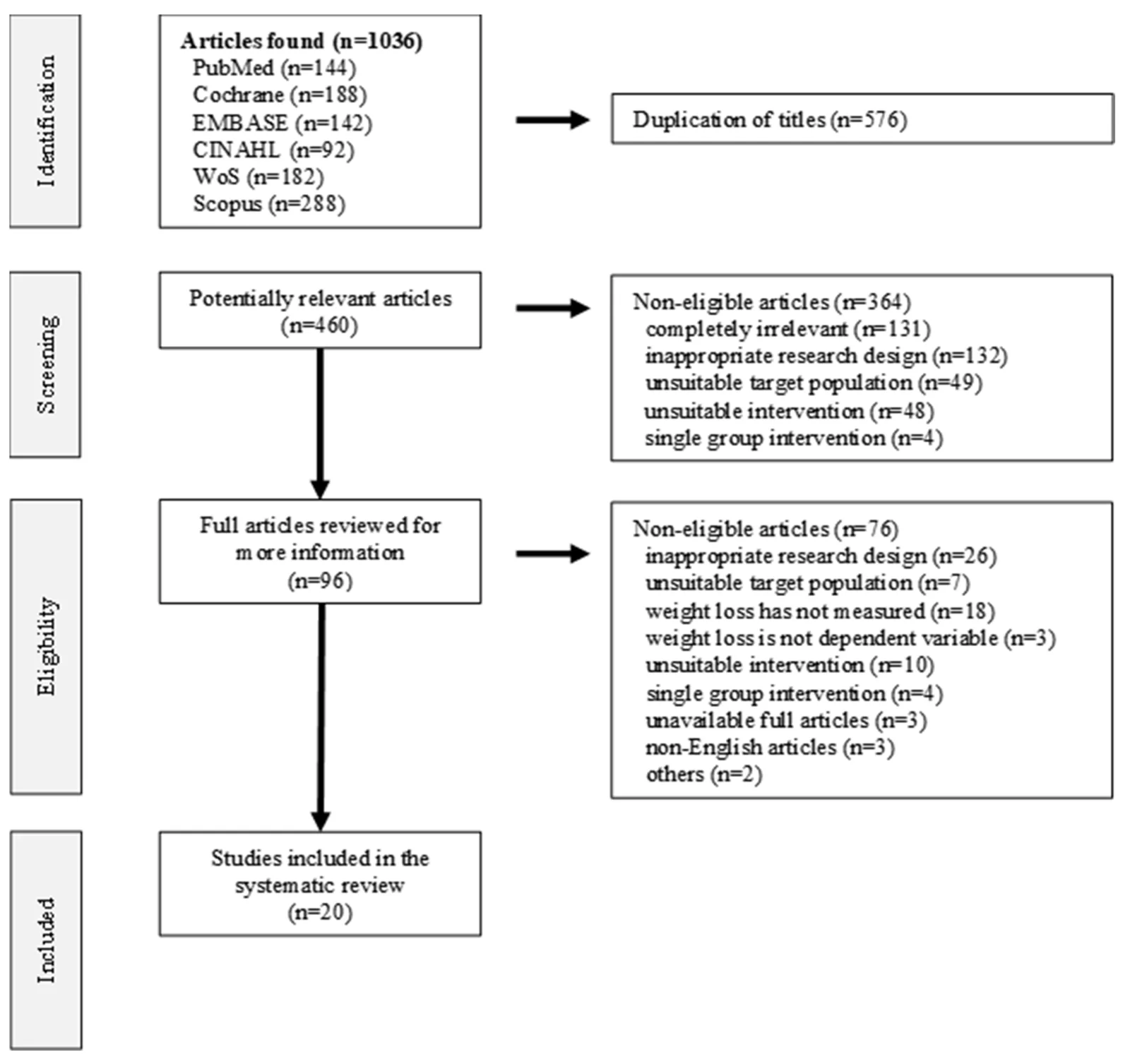
PRISMA flow diagram. Notes: The number of studies contributing to the quantitative syntheses varied by outcome. Fourteen studies contributed 15 comparisons to the primary meta-analysis of post-intervention body weight.

A total of 1036 records were identified through searches of six electronic databases. After removing 576 duplicate records, 460 articles remained for title and abstract screening. Of these, 364 records were excluded owing to irrelevance to the review question, inappropriate study design, unsuitable target population, ineligible intervention, or single-group design. Ninety-six full-text articles were subsequently assessed for eligibility. Following full-text review, 76 studies were further excluded for the following reasons: inappropriate research design (*n* = 26), unsuitable target population (*n* = 7), absence of weight loss outcomes (*n* = 18), weight loss not analyzed as a dependent variable (*n* = 3), ineligible intervention (*n* = 10), single-group design (*n* = 4), unavailable full text (*n* = 3), publication in a language other than English or Korean (*n* = 3), and others (*n* = 2). Ultimately, 20 articles met the inclusion criteria for qualitative synthesis. For the primary outcome of weight, studies that included multiple intervention arms (e.g., three-arm trials) were treated as separate comparisons for the intervention groups. Consequently, 15 comparisons were included in the meta-analysis (Figure 1).

### 3.2. Characteristics of the Included Studies

Among the 20 included studies, 14 were published before 2019, 16 reported ethical approval, and 12 reported funding support. Most of the included studies used a parallel-group randomized design. Across the 20 studies, 24 intervention comparisons were identified overall. Auricular acupressure was evaluated in 19 comparisons and auricular acupuncture in five. Seeds were used in 16 comparisons, magnetic beads in three, and needles in five. Fewer than six auricular points were used in 12 comparisons, and nine comparisons included adjunctive components such as massage, exercise, smartphone-based applications, dietary interventions, or thread-embedding techniques.

Sham, active, or adjunctive interventions were used in the control groups of 19 comparisons. Sixteen comparisons had intervention durations of eight weeks or longer, 11 involved at least 12 sessions, and four reported a session duration of at least five minutes. Delayed outcome assessment was reported in one comparison. Among the 15 comparisons contributing to the primary meta-analysis of post-intervention body weight, 10 had quality appraisal scores below the mean, and five had scores at or above the mean (Table 1 and Table S2).

### 3.3. Risk of Bias in Studies

The methodological quality of the 20 included studies was assessed using the Joanna Briggs Institute Checklist for RCTs. Across the 13 appraisal items, the mean quality score was 8.45 ± 2.26 out of a maximum of 13 points (Table 2). Item-level analysis indicated that several domains were consistently well reported. Follow-up completeness and appropriate handling of attrition (Q8), analysis according to randomized allocation (intention-to-treat principle; Q9), and reliability of outcome measurement (Q11) were reported in all 20 studies. Baseline comparability between treatment groups (Q3) and blinding of outcome assessors (Q6) were reported in all but one study. Conversely, domains related to selection and performance bias demonstrated lower reporting rates. Allocation concealment (Q2) and identical treatment of groups aside from the intervention of interest (Q7) were clearly reported in only five studies each. Notably, blinding of treatment providers (Q5) was reported in only one study, indicating a substantial potential risk of performance bias. Overall, while most included trials demonstrated adequate randomization procedures and reliable outcome assessment, limited reporting of allocation concealment and blinding procedures suggested a potential risk of bias.

### 3.4. Effect of Auricular Acupoint Stimulation on Weight

**Figure 2:**
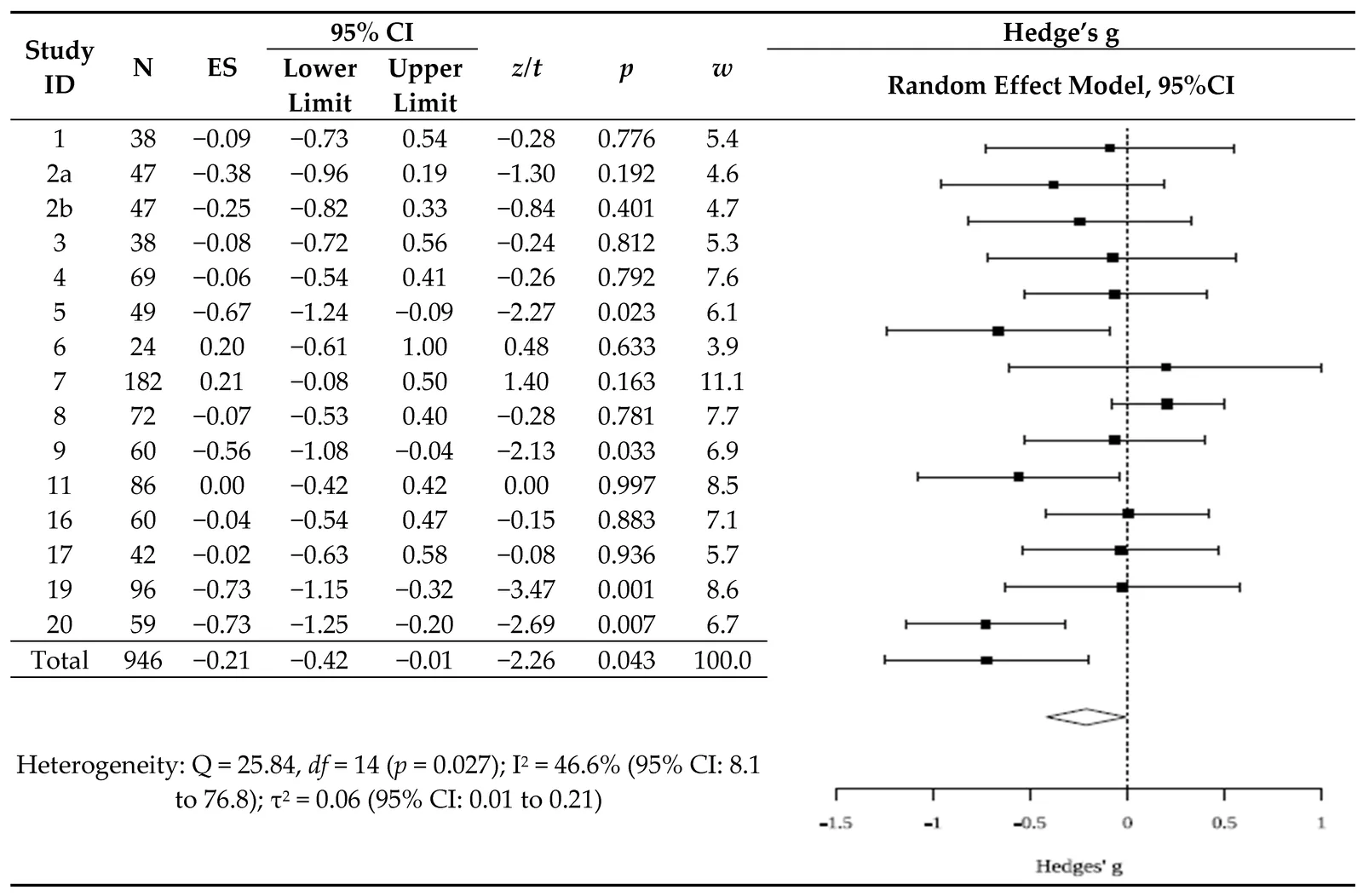
The effect of auricular acupoint stimulation on weight. Notes. The pooled estimate was obtained from the multilevel random-effects model, with the 95% CI and statistical inference based on small-sample-adjusted cluster-robust variance estimation (CR2). The diamond represents the pooled effect estimate and its 95% confidence interval.

Fourteen studies contributed 15 comparisons to the primary meta-analysis of post-intervention body weight (Figure 2). The multilevel random-effects model with CR2 inference yielded Hedges’ g = −0.21 (95% CI: −0.42 to −0.01, *p* = 0.043). Between-study heterogeneity was evident (Q = 25.84, df = 14, *p* = 0.027; I^2^ = 46.6%, 95% CI: 8.1–76.8%; τ^2^ = 0.06, 95% CI: 0.01–0.21). The 95% prediction interval ranged from −0.71 to 0.29.

Between-subgroup tests yielded *p* values below 0.05 for publication year (QM = 5.48, *p* = 0.019), funding status (QM = 4.84, *p* = 0.028), and quality appraisal score (QM = 5.11, *p* = 0.024). Within these categories, the effect estimates were −0.45 (95% CI: −0.78 to −0.13) for studies published in 2019 or later, −0.50 (95% CI: −0.80 to −0.20) for studies without funding support, and −0.46 (95% CI: −0.78 to −0.14) for studies with quality appraisal scores at or above the mean. The between-subgroup tests for the remaining characteristics had *p*-values above 0.05 (Table 3).

Univariate meta-regression analyses were conducted to examine associations between study characteristics and effect size. Publication year (β = −0.39, *p* = 0.019), funding status (β = 0.39, *p* = 0.028), and quality appraisal score (β = −0.39, *p* = 0.024) had *p* values below 0.05, whereas the remaining covariates did not (Table 4).

**Figure 3:**
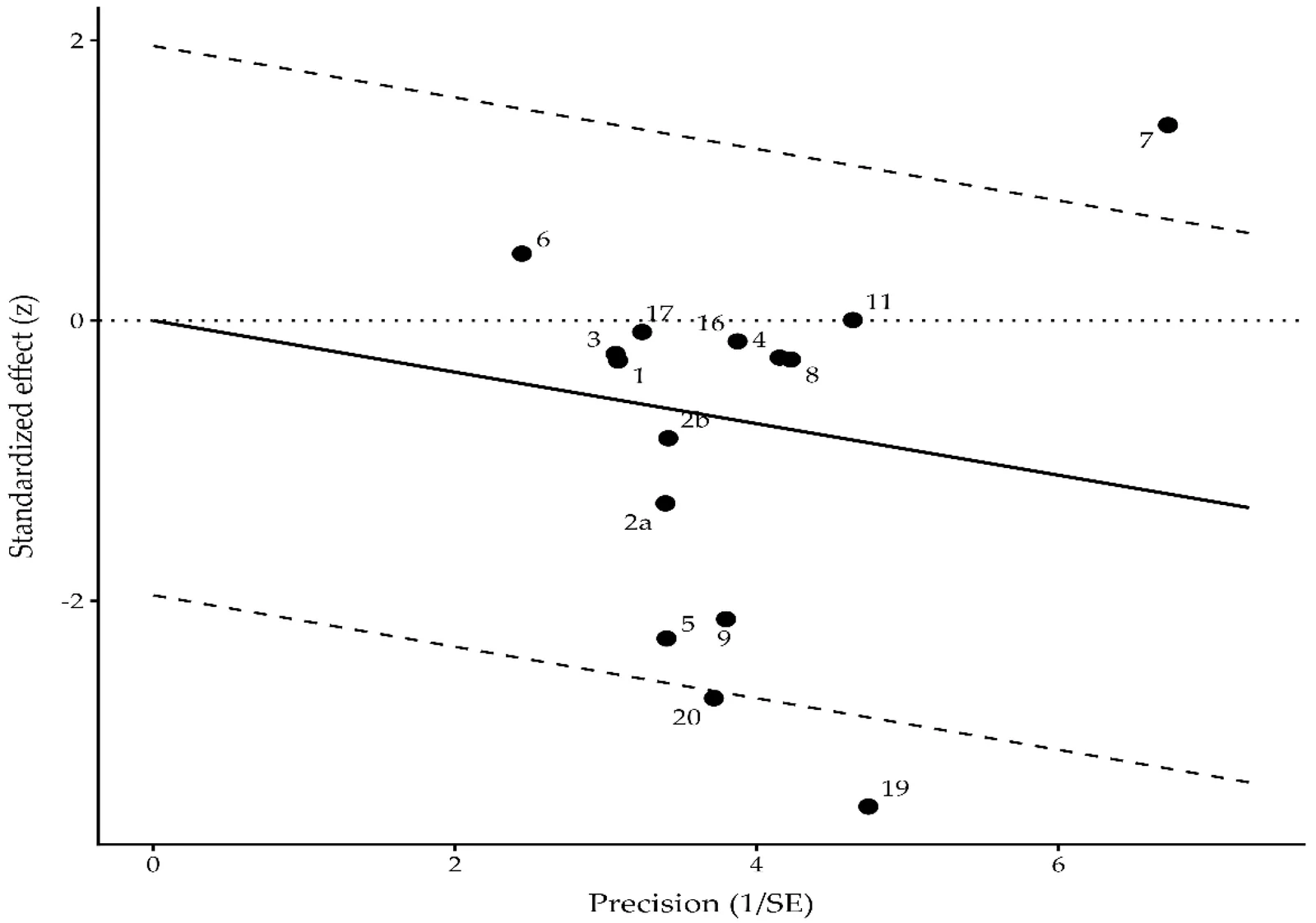
Galbraith plot of auricular acupoint stimulation on weight. Note. Points represent individual study comparisons; the solid line represents the regression line; the dashed lines indicate the 95% limits; and the horizontal dotted line represents the null effect (z = 0).

In the leave-one-out analysis, pooled effect estimates ranged from −0.27 to −0.16 and remained negative across all iterations. The 95% CI included the null value when Studies 2, 5, 9, 19, or 20 were omitted, whereas the remaining leave-one-out estimates had 95% CIs that excluded the null value (Table 5A). The Galbraith plot identified Studies 7 and 19 as outliers (Figure 3). In the multilevel model with CR2 inference, exclusion of these studies yielded an effect estimate of −0.22 (95% CI: −0.39 to −0.04, *p* = 0.021), compared with −0.21 (95% CI: −0.42 to −0.01, *p* = 0.043) when all 15 comparisons were retained (Table 5B). Across the model-based sensitivity analyses, the pooled estimates ranged from −0.22 to −0.18. The REML random-effects model yielded an estimate of −0.21 (95% CI: −0.40 to −0.03, *p* = 0.021), the DerSimonian–Laird model yielded −0.22 (95% CI: −0.40 to −0.03, *p* = 0.021), and the fixed-effect model yielded −0.18 (95% CI: −0.31 to −0.05, *p* = 0.006) (Table 5C).

### 3.5. Effects of Auricular Acupoint Stimulation on Secondary Outcomes

Among the secondary outcomes, the pooled estimate for change-from-baseline body weight was Hedges’ g = −1.42 (95% CI: −1.72 to −1.12, *p* < 0.001), with Q = 3.92 (df = 3, *p* = 0.270), I^2^ = 8.3%, and τ^2^ = 0.009 (Table 6). The estimates for BMI (Hedges’ g = −0.25, 95% CI: −0.49 to −0.01, *p* = 0.045) and leptin (Hedges’ g = −0.35, 95% CI: −0.69 to −0.02, *p* = 0.037) also had confidence intervals excluding the null value. For systolic blood pressure, the pooled estimate was positive (Hedges’ g = 0.41, 95% CI: 0.00 to 0.82, *p* = 0.049). Thus, nominally significant effects were observed for BMI, leptin, and systolic blood pressure, whereas the confidence intervals for the remaining secondary outcomes included the null value (Table 6).

### 3.6. Publication Bias

**Figure 4:**
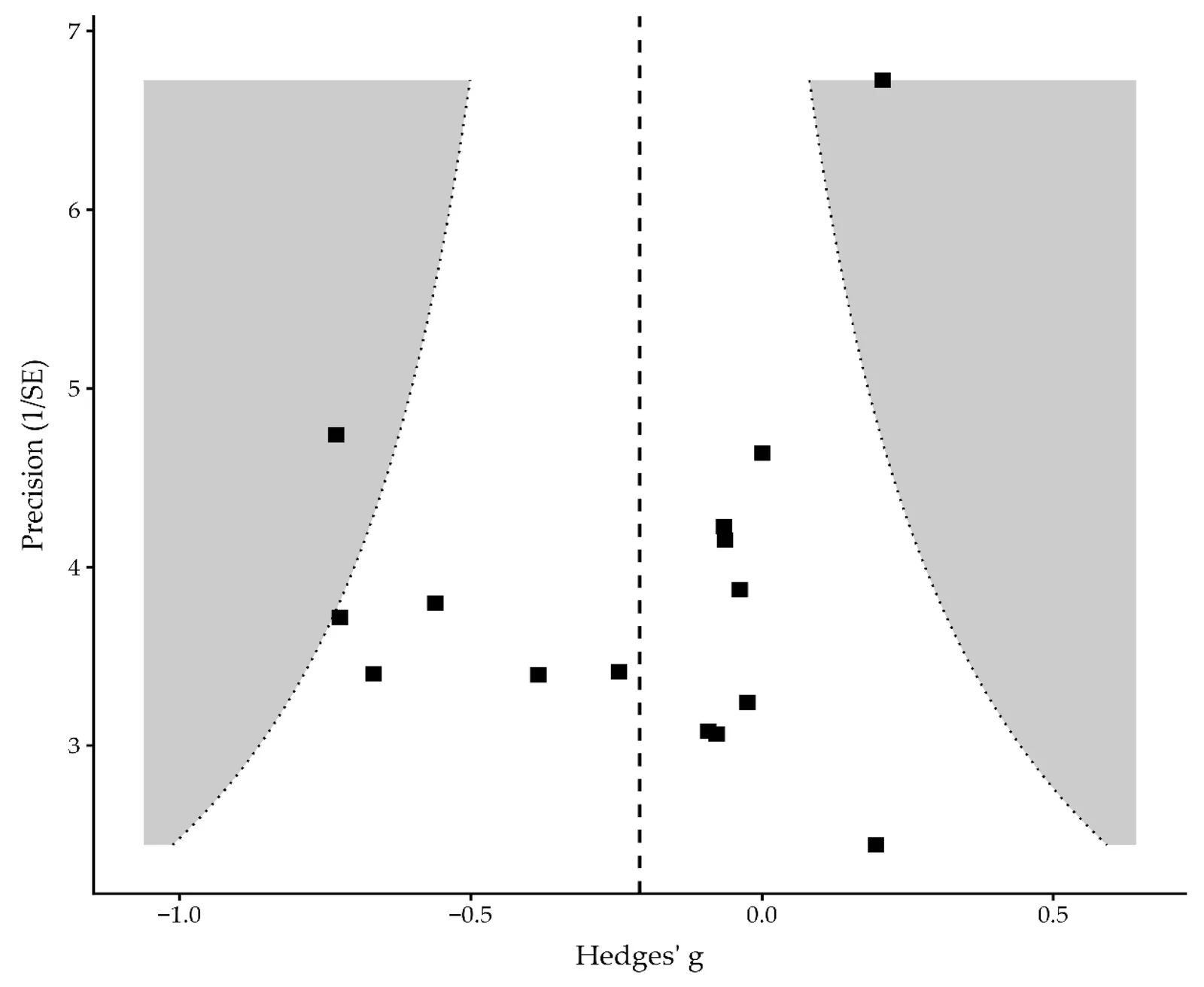
Funnel plot of auricular acupoint stimulation on weight reduction. Note. Black squares represent individual study comparisons; the vertical dashed line indicates the pooled effect estimate; the dotted curves indicate the pseudo 95% confidence limits; and the gray shaded areas represent regions outside these limits.

Potential publication bias and small-study effects were examined using funnel-plot inspection, Egger’s regression test, and the trim-and-fill method. Visual inspection of the funnel plot did not suggest pronounced asymmetry (Figure 4). Egger’s regression intercept was −0.04 (SE = 0.39, 95% CI: −0.81 to 0.72, *p* = 0.914), providing no statistical evidence of funnel-plot asymmetry. The trim-and-fill procedure did not estimate any additional studies, and the pooled estimate was unchanged (Hedges’ g = −0.21, 95% CI: −0.40 to −0.03) (Table 7). Taken together, these analyses did not indicate pronounced publication bias; however, publication bias could not be ruled out because the assessment was based on only 14 studies contributing 15 comparisons.

## 4. Discussion

This systematic review and meta-analysis evaluated the effects of auricular acupoint stimulation in adults aged 19 years or older with overweight or obesity. Auricular acupoint stimulation was associated with a small but statistically significant reduction in post-intervention body weight, although the prediction interval included the null value, indicating uncertainty regarding the consistency of the effect across settings. Among the secondary outcomes, nominally significant effects were observed for BMI, leptin, and systolic blood pressure. However, these findings should be considered exploratory because multiple secondary outcomes were examined without adjustment for multiplicity, and some outcomes were informed by relatively few studies. Accordingly, these findings should not be interpreted as definitive evidence of metabolic benefit.

Our findings align with and diverge from previous systematic reviews on auricular therapies for obesity. Hua et al. reported significant reductions in BMI, body weight, leptin, insulin, and HOMA-IR across 15 trials (*n* = 1333) examining multiple auricular stimulation modalities but found no significant effects on cholesterol or triglycerides [10]. In the present review, the secondary metabolic findings were also limited and should be interpreted cautiously. Our review specifically examined interventions in which auricular acupoint stimulation was the principal intervention. Co-interventions were not uniformly restricted across the included trials, and several studies combined auricular acupoint stimulation with dietary modification, exercise, lifestyle interventions, or other adjunctive components. Because the type and intensity of these co-interventions varied across studies, the independent effect of auricular acupoint stimulation could not be fully distinguished from the effects of the co-interventions. Such variation in co-intervention strategies may also have contributed to differences in effect estimates across studies and should be considered when interpreting the pooled findings.

Yeh et al. reported significant reductions in weight and BMI across 13 RCTs but noted substantial heterogeneity (I^2^ up to 88%) [11]. Similarly, moderate heterogeneity was observed in our primary analysis (I^2^ = 46.6%), suggesting variability in treatment protocols, participant characteristics, intervention duration, co-interventions, and outcome assessment. Kim et al. also found that acupuncture effects were significant only when combined with lifestyle modification [12], suggesting that co-interventions may contribute to variability in observed treatment effects. Huang et al. reported inconsistent results for auricular acupoint stimulation in individuals with overweight and obesity [13], while other reviews noted weight and BMI reductions but highlighted limitations such as small sample sizes, moderate study quality, and lack of standardized protocols [14,15,16]. The subgroup analyses also showed smaller effect sizes in more recent studies and in studies with higher JBI CAT scores. One possible explanation is that more recent studies may have employed more rigorous study designs, improved reporting practices, and better control of potential sources of bias, resulting in more conservative effect estimates. Similarly, higher JBI CAT scores may reflect greater adherence to methodological criteria that reduce the potential for inflated intervention effects. However, these findings should be interpreted cautiously because the subgroup analyses were based on a limited number of studies, and publication year and methodological characteristics may be interrelated. Our review extends the existing literature by examining metabolic biomarkers beyond anthropometric outcomes and by focusing on auricular acupoint stimulation as the intervention of interest. Several biological pathways have been proposed as possible mechanisms underlying the effects of auricular acupoint stimulation; however, these mechanisms were not directly evaluated in the present meta-analysis and should therefore be interpreted cautiously. From a neurophysiological perspective, auricular acupoint stimulation may act through stimulation of the auricular branch of the vagus nerve, which modulates parasympathetic activity and hypothalamic centers regulating appetite, satiety, and energy expenditure [10]. Previous research has suggested that auricular stimulation may influence metabolic regulation through vagal sensory pathways [10].

The significant reduction in leptin levels observed in our meta-analysis is particularly notable. Obesity is commonly characterized by elevated leptin levels and leptin resistance, in which hypothalamic sensitivity to leptin’s satiety signaling is impaired [48]. Acupuncture has been reported to improve leptin resistance via activation of the adenosine monophosphate-activated protein kinase signaling pathway, a key regulator of cellular energy homeostasis [17]. The reduction in leptin levels observed in our meta-analysis may warrant further investigation. Obesity is commonly characterized by elevated leptin levels and leptin resistance, in which hypothalamic sensitivity to leptin’s satiety signaling is impaired [48]. Acupuncture has been proposed to influence leptin-related signaling pathways [17]. Reduced circulating leptin could potentially reflect changes in leptin sensitivity or other metabolic pathways; however, these mechanisms were not directly assessed in the included studies.

The secondary metabolic findings should be interpreted cautiously. Although a nominally significant reduction in leptin was observed, the available evidence does not support a broader conclusion of improved metabolic health. Evidence for lipid-related outcomes was limited and inconsistent, and the present review did not provide comprehensive evidence across lipid parameters such as LDL-C, HDL-C, and triglycerides or clinical outcomes in populations with established metabolic or cardiovascular conditions, such as type 2 diabetes, metabolic syndrome, or cardiovascular disease. Therefore, these secondary findings should be regarded as exploratory rather than as evidence of comprehensive metabolic benefit.

Traditional Chinese medicine interventions have been proposed to influence obesity-related pathways involving appetite regulation, inflammation, and the brain–gut axis; however, these mechanisms vary substantially across intervention types and should not be directly attributed to auricular stimulation [48].

The discordance between changes in anthropometric measures and blood pressure outcomes may reflect the multifactorial regulation of blood pressure. Changes in body weight may not result in parallel or immediate changes in blood pressure, particularly during relatively short intervention periods, because blood pressure is also influenced by factors such as autonomic regulation, vascular resistance, fluid balance, baseline blood pressure, and concomitant treatments. However, these mechanisms were not directly evaluated in the included studies and should therefore be regarded as possible explanations rather than established mechanisms underlying the observed findings.

From a TCM perspective, auricular acupoint stimulation has also been interpreted within traditional concepts related to the regulation of energy balance. However, these traditional concepts were not operationalized or evaluated in the included studies and are therefore not used to explain the metabolic findings of the present review. Although the present review focused on the efficacy of auricular acupoint stimulation, safety and adverse events were not prespecified as review outcomes and were not systematically extracted. Therefore, the present findings should not be interpreted as evidence regarding safety, and no conclusions can be drawn regarding the safety profile of auricular acupoint stimulation.

### 4.1. Clinical Implications

Our findings have several clinical implications. The small effect observed for body weight suggests that auricular acupoint stimulation may have a modest role in weight management. Although nominally significant findings were observed for several secondary outcomes, including BMI, leptin, and systolic blood pressure, these results should be considered exploratory because of the potential for type I error arising from multiple comparisons and the limited number of studies contributing to some outcomes. Therefore, these secondary findings should not currently be considered definitive evidence of clinical or metabolic benefit.

Auricular acupoint stimulation may also have potential as an adjunctive therapy within comprehensive obesity management programs. Previous evidence suggests that acupuncture effects may be more pronounced when combined with lifestyle modification [12]. Consistent with this, systematic reviews have reported improvements in weight-related outcomes when acupuncture is used alongside lifestyle interventions [6,8]. However, further well-designed trials are needed to determine whether auricular acupoint stimulation provides additional benefit when combined with lifestyle interventions. Clinical interpretation should also consider differences in obesity severity and comorbidity burden, as the available evidence did not permit assessment of whether treatment effects differed across obesity categories.

### 4.2. Strengths of This Review

This systematic review has several methodological strengths. First, rigorous methods were applied, including duplicate independent screening, data extraction, and quality assessment, reducing the risk of error and bias. Second, we evaluated metabolic outcomes such as leptin, cholesterol, insulin resistance markers, and adipokines, extending beyond anthropometric measures to clinically relevant metabolic parameters. Finally, advanced statistical methods were used to explore heterogeneity and assess publication bias.

### 4.3. Limitations

Several limitations should be acknowledged. First, moderate heterogeneity was observed for the primary body-weight outcome, likely reflecting variation in treatment protocols, participant characteristics, concurrent interventions, and outcome assessment. In addition, the prediction interval included the null value, indicating uncertainty regarding the consistency of the effect across comparable settings. Second, most included studies had relatively small sample sizes, and several secondary outcomes were informed by relatively few studies, limiting the precision and robustness of the corresponding estimates. In addition, multiple secondary outcomes were analyzed without adjustment for multiplicity, increasing the potential for type I error. Therefore, nominally significant findings for secondary outcomes should be considered exploratory and require confirmation in future adequately powered studies. Third, follow-up durations were generally short (8–12 weeks), which restricts the assessment of long-term weight maintenance and sustained metabolic effects. Fourth, although visual inspection, Egger’s regression test, and the trim-and-fill analysis did not indicate pronounced publication bias, publication bias could not be ruled out because the assessment was based on only 14 studies contributing 15 comparisons. Fifth, although Korean-language studies were eligible for inclusion, Korean-specific bibliographic databases, such as OASIS and RISS, were not searched. Chinese- and Japanese-language databases, such as CNKI and CiNii, were also not included. Because studies of Traditional East Asian medicine may be indexed exclusively in these regional databases, relevant studies may have been missed. This restricted database and language coverage may have introduced retrieval- and language-related publication bias and may have affected the comprehensiveness and magnitude of the pooled estimates. Sixth, considerable variability and lack of standardization in treatment protocols—including acupoint selection, stimulation frequency, treatment duration, and stimulation devices—make it difficult to identify optimal treatment parameters. Seventh, the included populations varied in baseline adiposity (including weight and BMI), sex distribution, clinical setting, geographic region, and comorbidity status. These characteristics were not consistently reported or defined across studies, and the number of studies within potential subgroups was insufficient for reliable subgroup analyses. Therefore, residual population-related heterogeneity may remain, and the generalizability of the pooled estimates should be interpreted with caution. In addition, most trials were conducted in Asian populations, which may further limit the generalizability of the findings to other populations. Eighth, the included studies did not provide a sufficient number of clearly defined older-adult samples to permit age-specific pooled analyses; therefore, the findings should not be interpreted as evidence of effectiveness specifically in older adults. Finally, safety and adverse events were not prespecified as review outcomes and were not systematically extracted or analyzed; therefore, no conclusions regarding the safety profile of auricular acupoint stimulation can be drawn from this review.

### 4.4. Future Research Directions

The findings highlight several priorities for future research. Large, well-designed RCTs with adequate sample sizes are needed to provide more precise effect estimates. Future studies should adopt standardized treatment protocols and examine optimal parameters such as acupoint combinations, stimulation frequency, and treatment duration. Longer follow-up periods (≥ 6–12 months) are also required to evaluate the long-term sustainability of metabolic effects. Concurrent lifestyle interventions should be carefully measured and reported to distinguish the independent effects of auricular acupoint stimulation.

Additionally, studies should incorporate comprehensive metabolic assessments, including insulin sensitivity, inflammatory markers, adipokines, and body composition. Mechanistic studies using neuroimaging, autonomic function testing, and metabolomic approaches may help clarify underlying pathways. Finally, comparative effectiveness studies are necessary to position auricular acupoint stimulation relative to other complementary therapies and conventional obesity treatments.

## 5. Conclusions

This systematic review and meta-analysis found that auricular acupoint stimulation was associated with a small reduction in post-intervention body weight among adults aged 19 years or older with overweight or obesity. However, the prediction interval included the null value, indicating uncertainty regarding the consistency of this effect across settings. Nominally significant findings were observed for some secondary outcomes, including BMI, leptin, and systolic blood pressure; however, these findings should be considered exploratory because of multiple comparisons and the limited number of studies contributing to some outcomes. Further well-designed and adequately powered randomized controlled trials are needed to confirm these findings and determine their clinical relevance.

## Figures and Tables

**Table 1:** Descriptive summary of the included studies.

Study ID	Author (Year)	Country	IRB	Fund	Participants	Research Design	Intervention Type	Intervention Tool	Intervention Stimulus Points	EG Intervention	CG Intervention	Intervention Duration	Intervention Session	Intervention Time/Session	Outcome Measurement Time	Quality Score	Reported Outcomes
1	Yeh & Yeh (2008) [28]	China	Yes	Yes	38 non-obese healthy and obese volunteers (E: 19, C: 19)	Open parallel RCT	Acupressure	Magnetic beads	5 (Shenmen, mouth, stomach, endocrine points, small intestine)	Acupressure + massage	No	9 weeks	9 sessions	15 min	Immediately	8	BW, BF, BMI, WC, HC, WHR
2	Lien et al. (2012) [29]	Taiwan	Yes	No	77 women with obesity (E^a^: 24, E^b^: 24, C: 29)	Single- blind RCT	Ea: Acupuncture, E^b^: Acupressure	E^a^: Needle, Eb: Magnetic beads	4 (Shenmen, stomach, hunger, endocrine)	E^a^: Needle, E^b^: Magnetic beads	Sham acupuncture	4 weeks	12 sessions	Not reported	Immediately	8	BW, BMI, WC, HC, WHR, glucose, TG, TC, HDL-C, LDL-C, adiponectin, insulin, ghrelin, leptin, HOMA-IR, WHOQoL-BREF (physical, psychological, social, environmental)
3	Cayir et al. (2017) [30]	Turkey	Yes	Yes	38 women with obesity (E: 21, C: 17)	Single- blind RCT	Acupuncture	Needle	2 (anti-aggression, stomach)	Acupressure	Body acupuncture	12 weeks	6 sessions	1 min	Immediately	6	BW, BMI, BFP, WC, HC, AEs
4	Allison et al. (1995) [31]	USA	No	Yes	69 obese adults (E: 35, C: 34)	Double- blind RCT	Acupressure	Seeds	6 (not reported (illustrated in figure))	Acupressure + massage	Wrist acupressure	12 weeks	Not reported	2.5 min	Immediately	10	BW, BF/fat mass, SBP, DBP, side effects/AEs
5	Kim et al. (2014) [32]	Korea	Yes	Yes	49 female college students with overweight or obesity (E: 25, C: 24)	Single- blind RCT	Acupressure	Seeds	5 (Shenmen, mouth, stomach, endocrine, small intestine)	Acupressure	No	4 weeks	4 sessions	0.4 min	Immediately	9	BW, BMI, BF, WHR, self-efficacy
6	Mohammadi & Eskandari (2022) [33]	Iran	Yes	No	24 women with obesity and overweight (E: 12, C: 12)	Single- blind RCT	Acupressure	Seeds	6 (P6, P5, P4, P3, P2, P1)	Chinese acupressure + traditional Iranian medicine + aerobic exercise program	Traditional Iranian medicine + aerobic exercise program	6 weeks	Not reported	1 min	Immediately	5	BW, WHR, TG, HDL-C, LDL-C, SBP, DBP, blood sugar
7	Abdi et al. (2012) [34]	Iran	Yes	Yes	182 individuals with overweight and obesity (E: 93, C: 89)	Single- blind RCT	Acupressure	Seeds	6 (Shenmen, stomach, hunger, mouth, center of ear, Sanjiao)	Acupressure	Sham acupressure (without seeds)	6 weeks	12 sessions	0.3 min	Immediately	6	BW, BF, BMI, WC, HC, WHR, FBG, TC, TG, HDL-C, LDL-C, anti-Hsp27, anti-Hsp60, anti-Hsp65, anti-Hsp70, hs-CRP
8	Ching et al. (2012) [35]	Taiwan	Yes	Yes	72 hospitalized patients with obesity and schizophrenia (E: 33, C: 39)	Single- blind RCT	Acupressure	Seeds	4 (hunger, stomach, Shenmen, endocrine)	Acupressure	Surgical tape only	8 weeks	16 sessions	4 min	Immediately	6	BW, WC, BF
9	Khaki et al. (2019) [36]	Iran	Yes	Yes	60 single girls with polycystic ovary syndrome (E: 30, C: 30)	Single- blind RCT	Acupressure	Seeds, gold, silver, and magnetic beads	9 (endocrine, uterus, ovaries, lungs, kidneys, liver, adrenal, skin, and appetite)	Acupressure	Medication	8 weeks	10 sessions	Not reported	Delayed	7	Hirsutism score, hair loss, acne, menstrual irregularity, BW
10	Suen et al. (2019) [37]	Hong Kong	Yes	Yes	59 individuals with overweight (E^a^: 19, E^b^: 19, C: 21)	Single- blind RCT	Acupressure	Seeds	6 (Shenmen, stomach, endocrine, external nose, large intestine, forehead)	E^a^: Acupressure only, E^b^: Acupressure + smartphone app	No	8 weeks	16 sessions	Not reported	Immediately	11	BW, BMI, WC, HC, BF, visceral fat rating, leptin, adiponectin, fullness before meals
11	Darbandi et al. (2012) [38]	Iran	Yes	Yes	86 individuals with overweight or obesity (E: 43, C: 43)	Single- blind RCT	Acupressure	Seeds	6 (Shenmen, stomach, hunger, mouth, center of ear, Sanjiao)	Acupressure	Sham acupressure (without seeds)	6 weeks	2 sessions	Not reported	Immediately	8	BW, BMI, BFM, leptin
12	Yeh et al. (2015) [39]	Taiwan	Yes	No	70 patients with obesity (E: 36, C: 34)	Double- blind RCT	Acupressure	Seeds	4 (Shenmen, stomach, endocrine, hunger point)	Acupressure + electrical acupoint stimulation	Electrical acupoint stimulation at other site + pressure	10 weeks	1 session	4 min	Immediately	12	BMI, SBP, DBP, TC, TG, leptin, adiponectin
13	Song et al. (2015) [40]	China	No	No	45 patients with overweight and obesity with secondary hyperlipidemia (E^a^: 15, E^b^: 15, C: 15)	Single- blind RCT	Acupressure	Seeds	10 (heart, lung, liver, spleen, kidney, Sanjiao, small intestine, large intestine, gallbladder, stomach)	E^a^: Acupressure, E^b^: Acupressure + tai chi exercise	Tai chi exercise	26 weeks	52 sessions	150 min (E1) 7.5 min (E2 group)	Immediately	7	BMI, BF, TC, TG, LDL-C, HDL-C, apoB100, apoAI
14	Sun & Xu (1993) [41]	China	No	No	161 simple patients with obesity with or without hyperlipemia (E: 110, C: 51)	Single- blind RCT	Acupressure	Seeds	8 (mouth, esophagus, stomach, abdomen, hunger, Shenmen, lung, endocrine)	Acupressure + body acupuncture	Oenothera erythrosepalae olei capsulae	13 weeks	18 sessions	Not reported	Immediately	6	BW, overweight %, index of BW, BF, abdominal fat thickness, chest/abdomen/arm/thigh/calf circumferences, TC, TG, HDL-C, appetite, sleep, bowel movement
15	Yeo et al. (2014) [42]	Korea	Yes	Yes	58 individuals with obesity (E^a^: 22, E^b^: 21, C: 15)	Double- blind RCT	Acupuncture	Needle	5 (Shenmen, stomach, spleen, hunger, endocrine)	E^a^: Acupressure + needle acupuncture at 5 sites, E^b^: Acupressure + needle acupuncture at hunger point only	Sham acupuncture (without needle)	8 weeks	8 sessions	Not reported	Immediately	12	BMI, WC, BW, BFM, BF, SBP, DBP
16	He et al. (2012) [43]	China	Yes	Yes	60 Han Chinese women with primary obesity (E: 30, C: 30)	Single- blind RCT	Acupressure	Seeds	6 (hunger, stomach, spleen, large intestine, endocrine, Shenmen)	Acupressure	No	4 weeks	9 sessions	3 min	Immediately	7	BW, BMI, WC
17	Ben-Arie et al. (2025) [44]	Taiwan	Yes	Yes	44 individuals with abdominal obesity (E: 21, C: 23)	Single- blind RCT	Acupressure	Seeds	3 (Shenmen, stomach, endocrine)	Acupressure + abdominal catgut embedding	Abdominal catgut embedding	12 weeks	12 sessions	5 min	Immediately	12	BW, BMI, BMR, BF%, WHR, TG, TC
18	Trinh et al. (2024) [45]	Vietnam	No	No	66 individuals with overweight or obesity (E: 33, C:33)	Double- blind RCT	Acupuncture	Needle	5 (Shenmen, stomach, spleen, endocrine, hunger points)	Acupressure + thread embedding	Sham thread embedding	8 weeks	12 sessions	Not reported	Immediately	11	BW, BMI, WC, HC, WHR, FCQ-Tr score, food craving VAS score, AEs
19	Zhao et al. (2025) [46]	China	Yes	No	96 individuals with obesity (E: 48, C: 48)	Single- blind RCT	Acupressure	Seeds	9 (Shenmen, Sanjiao, hunger point, thirst point, large intestine, endocrine, spleen, lung, stomach)	Acupressure + acupoint embedding therapy	Traditional acupoint therapy	30 days	10 sessions	Not reported	Immediately	8	BW, BMI, WC, TC, TG, LDL-C, HDL-C, HOMA-IR, leptin, hs-CRP, BF, BMR, TCM syndrome scores, AEs
20	Lee et al. (2026) [47]	Korea	Yes	No	59 individuals with BMI: 25–34 (E: 30, C: 29)	Single- blind RCT	Acupressure	Seeds	5 (Shenmen, heart, stomach, endocrine, hunger points)	Acupressure	Sham	8 weeks	8 sessions	2 min	Immediately	9	BW, BMI, WC, BFM, BF, PSQI-K, total sleep time, sleep efficiency, sleep latency, awakenings, light sleep, deep sleep, REM sleep

Notes. EG, intervention group; CG, control group; BW, body weight; BMI, body mass index; BF, body fat; BFP, body fat percentage; BFM, body fat mass; WC, waist circumference; HC, hip circumference; WHR, waist-to-hip ratio; FBG, fasting blood glucose; TC, total cholesterol; TG, triglycerides; HDL-C, high-density lipoprotein cholesterol; LDL-C, low-density lipoprotein cholesterol; SBP, systolic blood pressure; DBP, diastolic blood pressure; HOMA-IR, homeostasis model assessment of insulin resistance; hs-CRP, high-sensitivity C-reactive protein; Hsp, heat shock protein; apoB100, apolipoprotein B100; apoAI, apolipoprotein A-I; BMR, basal metabolic rate; WHOQoL-BREF, World Health Organization Quality of Life-BREF; FCQ-Tr, Food Cravings Questionnaire-Trait-reduced; VAS, visual analog scale; TCM, traditional Chinese medicine; PSQI-K, Korean version of the Pittsburgh Sleep Quality Index; REM, rapid eye movement; AEs, adverse events.

**Table 2:** Quality appraisal of the included studies.

Study ID	Joanna Briggs Institute of Critical Appraisal Tools Checklist for Randomized Controlled Trials													Total Score
	Q1	Q2	Q3	Q4	Q5	Q6	Q7	Q8	Q9	Q10	Q11	Q12	Q13	
1	1	0	1	0	0	1	0	1	1	1	1	0	1	8
2	1	0	1	1	0	0	0	1	1	1	1	0	1	8
3	0	0	1	0	0	1	0	1	1	1	1	0	0	6
4	1	0	1	1	0	1	0	1	1	1	1	1	1	10
5	1	0	1	0	0	1	0	1	1	1	1	1	1	9
6	0	0	1	0	0	1	0	1	1	0	1	0	0	5
7	0	0	1	0	0	1	0	1	1	1	1	0	0	6
8	0	0	1	0	0	1	0	1	1	1	1	0	0	6
9	1	0	1	0	0	1	0	1	1	0	1	0	1	7
10	1	1	1	0	0	1	1	1	1	1	1	1	1	11
11	1	0	1	0	0	1	0	1	1	1	1	0	1	8
12	1	1	1	1	1	1	0	1	1	1	1	1	1	12
13	1	0	1	0	0	1	0	1	1	0	1	0	1	7
14	0	0	1	1	0	1	0	1	1	0	1	0	0	6
15	1	1	1	1	0	1	1	1	1	1	1	1	1	12
16	0	0	1	0	0	1	1	1	1	1	1	0	0	7
17	1	1	1	1	0	1	1	1	1	1	1	1	1	12
18	1	1	0	1	0	1	1	1	1	1	1	1	1	11
19	1	0	1	0	0	1	0	1	1	1	1	1	1	9
20	1	0	1	0	0	1	0	1	1	1	1	1	1	9
Total	14	5	19	7	1	19	5	20	20	16	20	9	14	8.45 ± 2.26

Notes. Q1. Was true randomization used for assignment of participants to treatment groups? Q2. Was allocation to treatment groups concealed? Q3. Were treatment groups similar at baseline? Q4. Were participants blind to treatment assignment? Q5. Were those delivering treatment blind to treatment assignment? Q6. Were outcomes assessors blind to treatment assignment? Q7. Were treatment groups treated identically other than the intervention of interest? Q8. Was follow-up complete and if not, were differences between groups in terms of their follow-up adequately described and analyzed? Q9. Were participants analyzed in the groups to which they were randomized? Q10. Were outcomes measured in the same way for treatment groups? Q11. Were outcomes measured in a reliable way? Q12. Was appropriate statistical analysis used? Q13. Was the trial design appropriate, and any deviations from the standard randomized controlled trial design (individual randomization, parallel groups) accounted for in the conduct and analysis of the trial?

**Table 3:** Subgroup analysis regarding effect of auricular acupoint stimulation on weight by study characteristics.

Characteristics	Category	K	Study ID	ES	95% CI		z	*p*	*I* ^2^	QM	*p* for Subgroup Difference
					Lower Limit	Upper Limit					
Year	<2019	10	1, 2 ^a^, 2 ^b^, 3, 4, 5, 7, 8, 11, 16	−0.07	−0.24	0.10	−0.80	0.422	15.3	5.48	0.019
	≥2019	5	6, 9, 17, 19, 20	−0.45	−0.78	−0.13	−2.72	0.006	42.7		
IRB	No	1	4	−0.06	−0.54	0.41	−0.26	0.792		0.20	0.655
	Yes	14	1, 2 ^a^, 2 ^b^, 3, 5, 6, 7, 8, 9, 11, 16, 17, 19, 20	−0.23	−0.42	−0.03	−2.30	0.021	47.7		
Fund	No	6	2 ^a^, 2 ^b^, 6, 11, 19, 20	−0.50	−0.80	−0.20	−3.30	0.001	19.7	4.84	0.028
	Yes	9	1, 3, 4, 5, 7, 8, 9, 16, 17	−0.09	−0.27	0.09	−1.03	0.305	26.1		
Number of participants	<60	8	1, 2 ^a^, 2 ^b^, 3, 5, 6, 17, 20	−0.30	−0.54	−0.06	−2.47	0.013	11.8	0.44	0.506
	≥60	7	4, 7, 8, 9, 11, 16, 19	−0.16	−0.43	0.10	−1.21	0.226	61.8		
Intervention type	Acupressure	13	1, 2 ^b^, 4, 5, 6, 7, 8, 9, 11, 16, 17, 19, 20	−0.21	−0.41	−0.02	−2.13	0.033	50.7	0.02	0.895
	Acupuncture	2	2 ^a^, 3	−0.25	−0.68	0.18	−1.13	0.260	0.0		
Intervention tool	Seeds	11	4, 5, 6, 7, 8, 9, 11, 16, 17, 19, 20	−0.22	−0.45	0.00	−1.94	0.053	58.0	0.09	0.958
	Magnetic beads	2	1, 2 ^b^	−0.18	−0.60	0.25	−0.81	0.416	0.0		
	Needle	2	2 ^a^, 3	−0.25	−0.68	0.18	−1.13	0.260	0.0		
Intervention stimulus points	<6	8	1, 2 ^a^, 2 ^b^, 3, 5, 8, 17, 20	−0.29	−0.52	−0.07	−2.62	0.009	9.5	0.57	0.451
	≥6	7	4, 6, 7, 9, 11, 16, 19	−0.15	−0.43	0.13	−1.03	0.303	62.8		
EG adjunct intervention	No	10	2 ^a^, 2 ^b^, 3, 5, 7, 8, 9, 11, 16, 20	−0.22	−0.44	0.01	−1.91	0.056	47.9	0.00	0.979
	Yes	5	1, 4, 6, 17, 19	−0.21	−0.55	0.14	−1.15	0.250	48.3		
Control group intervention	No intervention	3	1, 5, 16	−0.26	−0.66	0.14	−1.27	0.203	32.1	0.06	0.813
	Sham or other intervention	12	2 ^a^, 2 ^b^, 3, 4, 6, 7, 8, 9, 11, 17, 19, 20	−0.21	−0.42	0.00	−1.93	0.054	50.0		
Intervention duration	<8 weeks	8	2 ^a^, 2 ^b^, 5, 6, 7, 11, 16, 19	−0.20	−0.48	0.08	−1.41	0.158	59.0	0.05	0.815
	≥8 weeks	7	1, 3, 4, 8, 9, 17, 20	−0.24	−0.46	−0.02	−2.11	0.035	15.1		
Intervention session	Not reported or <12 sessions	10	1, 3, 4, 5, 6, 9, 11, 16, 19, 20	−0.30	−0.52	−0.08	−2.69	0.007	42.1	2.24	0.135
	≥12 sessions	5	2 ^a^, 2 ^b^, 7, 8, 17	0.00	−0.24	0.24	0.02	0.982	15.3		
Intervention time/session	Not reported or <5 min	13	2 ^a^, 2 ^b^, 3, 4, 5, 6, 7, 8, 9, 11, 16, 19, 20	−0.22	−0.41	−0.03	−2.28	0.023	48.2	0.09	0.761
	≥5 min	2	1, 17	−0.09	−0.73	0.54	−0.28	0.776			
Outcome measurement time	Immediately	14	1, 2 ^a^, 2 ^b^, 3, 4, 5, 6, 7, 8, 11, 16, 17, 19, 20	−0.20	−0.40	0.00	−1.98	0.048	47.8	0.17	0.677
	Delayed	1	9	−0.32	−0.84	0.21	−1.19	0.235	42.7		
Quality appraisal score	<Mean	10	1, 2 ^a^, 2 ^b^, 3, 6, 7, 8, 9, 11, 16	−0.06	−0.24	0.12	−0.67	0.504	16.3	5.11	0.024
	≥Mean	5	4, 5, 17, 19, 20	−0.46	−0.78	−0.14	−2.80	0.005	48.6		

Notes. Subgroup-specific pooled estimates were obtained using random-effects REML models with adjustment for shared control groups by dividing the control-group sample size across the relevant comparisons. Formal tests for subgroup differences were conducted using omnibus moderator tests (QM) from multilevel random-effects meta-regression models. Superscripts a and b distinguish separate intervention comparisons within the same study; K, number of comparisons; N, number of participants; ES, effect size; CI, confidence interval; QM, Q statistic for the test of subgroup differences; IRB, institutional review board; EG, intervention group; CG, control group.

**Table 4:** Meta-regression analysis evaluating effect of auricular acupoint stimulation on weight.

Covariates (Ref.)	Coefficients (β)	SE	95% CI		*z*	*p*
			Lower Limit	Upper Limit		
Year (≥2019, Ref.: <2019)	−0.39	0.17	−0.72	−0.06	−2.34	0.019
IRB (Yes, Ref.: No)	−0.16	0.36	−0.87	0.55	−0.45	0.655
Fund (Yes, Ref.: No)	0.39	0.18	0.04	0.73	2.20	0.028
Participants (≥60, Ref.: <60)	0.13	0.19	−0.25	0.50	0.67	0.506
Intervention type (Acupuncture, Ref.: Acupressure)	−0.04	0.28	−0.58	0.50	−0.13	0.895
Intervention tool (Seeds, Ref.: Needle)	0.08	0.30	−0.51	0.66	0.26	0.794
Intervention tool (Magnetic beads, Ref.: Needle)	−0.01	0.30	−0.60	0.58	−0.03	0.975
Intervention stimulus points (≥6, Ref.: <6)	0.14	0.19	−0.23	0.51	0.75	0.451
EG adjunct intervention (Yes, Ref.: No)	0.01	0.21	−0.40	0.41	0.03	0.979
CG intervention (Yes, Ref.: No)	0.06	0.25	−0.42	0.54	0.24	0.813
Intervention duration (≥8 weeks, Ref.: <8 weeks)	−0.05	0.19	−0.42	0.33	−0.23	0.815
Intervention session (≥12 sessions, Ref.: Not reported or <12 sessions)	0.28	0.18	−0.09	0.64	1.50	0.135
Intervention time/session (≥5 min, Ref.: Not reported or <5 min)	0.13	0.42	−0.70	0.95	0.30	0.761
Outcome measurement time (Delayed, Ref.: Immediately)	−0.12	0.29	−0.68	0.44	−0.42	0.677
Quality appraisal score (≥Mean, Ref.: <Mean)	−0.39	0.17	−0.72	−0.05	−2.26	0.024

Notes. Univariate meta-regression analyses were conducted using multilevel random-effects models with REML estimation to account for multiple comparisons nested within studies. Regression coefficients (β) represent the estimated change in Hedges’ g relative to the reference category. Ref., reference; SE, standard error; CI, confidence interval; IRB, institutional review board; EG, intervention group; CG, control group.

**Table 5:** Sensitivity test of the effect of auricular acupoint stimulation on weight.

(**A**). Leave-one-out sensitivity analysis								
Omitted study ID	Author (year)	K	ES	95% CI		*t*	*df*	*p*
				Lower limit	Upper limit			
1	Yeh & Yeh (2008) [28]	13	−0.22	−0.44	−0.00	−2.23	11.35	0.047
2	Lien et al. (2012) [29]	13	−0.20	−0.43	0.02	−2.00	11.33	0.070
3	Cayir/Yasemin et al. (2017) [30]	13	−0.22	−0.44	−0.00	−2.24	11.35	0.046
4	Allison et al. (1995) [31]	13	−0.23	−0.45	−0.00	−2.24	11.29	0.046
5	Kim et al. (2014) [32]	13	−0.18	−0.39	0.03	−1.92	11.19	0.081
6	Mohammadi & Eskandari (2022) [33]	13	−0.23	−0.44	−0.02	−2.38	11.46	0.036
7	Abdi et al. (2012) [34]	13	−0.27	−0.46	−0.07	−3.03	11.23	0.011
8	Ching et al. (2012) [35]	13	−0.23	−0.45	−0.00	−2.24	11.29	0.046
9	Khaki et al. (2019) [36]	13	−0.19	−0.40	0.03	−1.92	11.20	0.081
11	Darbandi et al. (2012) [38]	13	−0.23	−0.45	−0.01	−2.32	11.28	0.040
16	He et al. (2012) [43]	13	−0.23	−0.45	−0.01	−2.27	11.28	0.044
17	Ben-Arie et al. (2025) [44]	13	−0.22	−0.44	−0.01	−2.27	11.32	0.044
19	Zhao et al. (2025) [46]	13	−0.16	−0.35	0.04	−1.77	10.86	0.104
20	Lee et al. (2026) [47]	13	−0.17	−0.38	0.03	−1.86	11.09	0.089
(**B**). Sensitivity analysis after excluding outliers identified from the Galbraith plot								
Study ID		K	ES	95% CI		*t*	*df*	*p*
				Lower limit	Upper limit			
Original		15	−0.21	−0.42	−0.01	−2.26	12.21	0.043
Exclude Galbraith outliers (Studies 7 and 19)		13	−0.22	−0.39	−0.04	−2.73	9.83	0.021
(**C**). Sensitivity analysis by meta-analytic model								
Model (estimate)		K	ES	95% CI		z	*p*	
				Lower limit	Upper limit			
Random effect model (REML)		15	−0.21	−0.40	−0.03	−2.31	0.021	
Random effect model (DerSimonian–Laird)		15	−0.22	−0.40	−0.03	−2.31	0.021	
Fixed effect model		15	−0.18	−0.31	−0.05	−2.74	0.006	

Notes. Original denotes the primary multilevel random-effects analysis of all 15 comparisons using study-clustered CR2 inference. Leave-one-out and outlier-exclusion analyses were conducted using the same multilevel random-effects model with study-clustered CR2 inference. Analyses in section C used conventional comparison-level models, with shared control-group sample sizes divided across the relevant comparisons. K, number of analysis sets; ES, effect size; CI, confidence interval; REML, restricted maximum likelihood.

**Table 6:** Effect of auricular acupoint stimulation on secondary outcomes.

Variables	K	Study ID	N	ES	95% CI		z	*p*	Q/df	*p* for Q	I^2^	τ^2^
					Lower Limit	Upper Limit						
Body-weight change	4	14, 15 ^a^, 15 ^b^, 18	300	−1.42	−1.72	−1.12	−9.28	<0.001	3.92/3	0.270	8.3	0.01
BMI	13	1, 2 ^a^, 2 ^b^, 3, 5, 7, 11, 13 ^a^, 13 ^b^, 16, 17, 19, 20	804	−0.25	−0.49	−0.01	−2.01	0.045	28.22/12	0.005	57.5	0.10
WHR	7	1, 2 ^a^, 2 ^b^, 5, 6, 7, 17	429	−0.03	−0.22	0.15	−0.36	0.719	3.49/6	0.745	0.0	0.00
BF	12	1, 3, 4, 5, 7, 8, 11, 13 ^a^, 13 ^b^, 17, 19, 20	791	−0.28	−0.57	0.01	−1.88	0.060	43.54/11	<0.001	74.7	0.17
WC	9	1, 2 ^a^, 2 ^b^, 3, 7, 8, 16, 19, 20	639	−0.21	−0.48	0.06	−1.55	0.121	18.26/8	0.019	56.2	0.09
HC	5	1, 2 ^a^, 2 ^b^, 3, 7	352	−0.08	−0.29	0.13	−0.71	0.480	3.78/4	0.436	0.0	0.00
SBP	2	4, 6	93	0.41	0.00	0.82	1.97	0.049	0.05/1	0.820	0.0	0.00
DBP	2	4, 6	93	0.40	−0.01	0.81	1.91	0.056	0.55/1	0.460	0.0	0.00
Leptin	5	2 ^a^, 2 ^b^, 11, 12, 19	346	−0.35	−0.69	−0.02	−2.09	0.037	7.33/4	0.120	45.4	0.07
FBG	4	2 ^a^, 2 ^b^, 6, 7	300	0.01	−0.23	0.25	0.07	0.943	0.75/3	0.861	0.0	0.00
TC	8	2 ^a^, 2 ^b^, 7, 12, 13a, 13 ^b^, 17, 19	546	−0.55	−1.14	0.04	−1.83	0.067	50.40/7	<0.001	86.1	0.49
TG	9	2 ^a^, 2 ^b^, 6, 7, 12, 13a, 13 ^b^, 17, 19	570	−0.42	−0.85	0.01	−1.93	0.053	39.57/8	<0.001	79.8	0.27

Notes. Superscripts a and b distinguish separate intervention comparisons within the same study. K, number of comparisons; N, number of participants; ES, effect size (Hedges’ g); CI, confidence interval; I^2^, heterogeneity statistic; τ^2^, between-study variance; BMI, body mass index; WHR, waist-to-hip ratio; BF, body fat; WC, waist circumference; HC, hip circumference; SBP, systolic blood pressure; DBP, diastolic blood pressure; FBG, fasting blood glucose; TC, total cholesterol; TG, triglycerides.

**Table 7:** Publication bias test of effect of auricular acupoint stimulation on weight.

Egger’s Regression Test	Coeff	SE	95% CI		z	*p*
			Lower Limit	Upper Limit		
Intercept	−0.04	0.39	−0.81	0.72	−0.11	0.914
Slope	−0.65	1.43	−3.45	2.16	−0.45	0.651
Trim-and-fill method	K	Hg	95% CI		z	*p*
			Lower limit	Upper limit		
Original	15	−0.21	−0.40	−0.03	−2.30	0.022
Trim-and-fill correction	15	−0.21	−0.40	−0.03	−2.30	0.022

Notes: Egger’s regression test and trim-and-fill analysis used a conventional random-effects REML model rather than the primary multilevel model with CR2 inference. SE: standard error, CI: confidence interval, K: number of analysis sets. Publication-bias analyses were based on a conventional random-effects model with adjustment for the shared control group, whereas the primary pooled effect was estimated using a multilevel random-effects model with small-sample-adjusted cluster-robust variance estimation (CR2). The intercept term was used to assess funnel-plot asymmetry.

## Data Availability

No new data were created or analyzed in this study. Data sharing is not applicable to this article.

## Supplementary Materials

The following supporting information can be downloaded at: https://www.mdpi.com/article/10.3390/healthcare14193336/s1, Table S1: Search strategy used to retrieve studies; Table S2: Full-text articles excluded after eligibility assessment and reasons for exclusion.

## Abbreviations

The following abbreviations are used in this manuscript:

BMI	Body mass index
CIs	Confidence intervals
HOMA-IR	Homeostatic model assessment of insulin resistance
REML	Restricted maximum likelihood
RCTs	Randomized controlled trials
TCM	Traditional Chinese medicine
