# Supplementary material for: Effect of Auricular Acupoint Stimulation on Weight in Adults with Overweight or Obesity: A Systematic Review and Meta-Analysis
Source: Healthcare (Basel). 2026 Oct 6;14(19):3336. doi: 10.3390/healthcare14193336 (PMC13665510; doi:10.3390/healthcare14193336)
Supplement: Supplementary file 1 [file healthcare-14-03336-s001.zip › healthcare-4499095-supplementary.pdf]

Supplementary S1.

**Table S1.** Search strategy used to retrieve studies

| Database | Search setting | Result (December 21, 2023, to March 21, 2024) | Result (December 10, 2025, to February 5, 2026) |
|---|---|---|---|
| PubMed | ( ( ( ( ( “Ear”[Mesh] OR “ear”[All Fields] OR “auricular”[All Fields] OR “auricule”[All Fields] ) AND ( “Acupressure”[Mesh] OR “acupress*”[All Fields] OR “finger press*”[All Fields] OR “point press*”[All Fields] OR “pressure bean*”[All Fields] OR “point taping”[All Fields] OR “point sticking”[All Fields] OR “ear seed*”[All Fields] OR “point seed*”[All Fields] OR “seed press*”[All Fields] OR “seed embedding”[All Fields] OR “acupoint*”[All Fields] OR “Shiatsu”[All Fields] OR “Zhi Ya”[All Fields] ) ) OR ( “ear pressing”[All Fields] OR “auricular pressing”[All Fields] OR “auriculoacupressure”[All Fields] OR “auriculoacupoint*”[All Fields] OR “auricular point*”[All Fields] ) OR ( “Acupuncture, Ear”[Mesh] OR ( ( “Ear”[Mesh] OR “ear”[All Fields] OR “auricular”[All Fields] OR “auricule”[All Fields] ) AND “acupunctur*”[All Fields] ) OR “auriculoacupuncture”[All Fields] ) OR ( “Auriculotherapy”[Mesh] OR “auriculotherapy”[All Fields] OR “auricular therapy”[All Fields] ) ) AND ( “Obesity”[Mesh] OR “obesity”[All Fields] OR “obese”[All Fields] OR “Body Weight”[Mesh] OR “Weight Loss”[Mesh] OR “Weight Gain”[Mesh] OR “weight”[All Fields] OR “overweight*”[All Fields] OR “Body Mass Index”[Mesh] OR “Waist Circumference”[Mesh] OR “Waist-Hip Ratio”[Mesh] OR “Skinfold Thickness”[Mesh] OR “body mass”[All Fields] OR “body fat”[All Fields] OR “waist circumference”[All Fields] OR “hip circumference”[All Fields] OR “waist hip ratio”[All Fields] OR “waist to hip ratio”[All Fields] OR “skinfold thickness”[All Fields] ) ) NOT ( Animals[Mesh] NOT “Humans”[Mesh] ) ) NOT ( (“Infant”[Mesh] OR “Child”[Mesh] OR “Adolescent”[Mesh]) NOT “Adult”[Mesh] ) | 118 | 26 |
| Cochrane | ( ( ( (((MeSH descriptor: [Ear] explode all trees OR (“ear” OR “auricular” OR “auricule”):ti,ab,kw) AND (MeSH descriptor: [Acupressure] explode all trees OR (acupress* OR (finger NEXT press*) OR (point NEXT press*) OR (pressure NEXT bean?) OR “point taping” OR “point sticking” OR (ear NEXT seed?) OR (point NEXT seed?) OR (seed NEXT press*) OR “seed embedding” OR acupoint* OR “Shiatsu” OR “Zhi Ya”):ti,ab,kw)) OR (“ear pressing” OR “auricular pressing” OR “auriculoacupressure” OR auriculoacupoint* OR (auricular NEXT point*)):ti,ab,kw) OR (MeSH descriptor: [Acupuncture, Ear] explode all trees OR ((MeSH descriptor: [Ear] explode all trees OR (“ear” OR “auricular” OR “auricule”):ti,ab,kw) AND (acupunctur*):ti,ab,kw) OR (“auriculoacupuncture”):ti,ab,kw) OR (MeSH descriptor: [Auriculotherapy] explode all trees OR (“auriculotherapy” OR “auricular therapy”):ti,ab,kw) ) AND ( (MeSH descriptor: [Obesity] explode all trees OR (“obesity” OR | 166 | 22 |

| | | | |
|---|---|---|---|
| | "obese"):ti,ab,kw) OR (MeSH descriptor: [Body Weight] explode all trees OR MeSH descriptor: [Weight Loss] explode all trees OR MeSH descriptor: [Weight Gain] explode all trees OR ("weight" OR overweight*):ti,ab,kw) OR (MeSH descriptor: [Body Mass Index] explode all trees OR MeSH descriptor: [Waist Circumference] explode all trees OR MeSH descriptor: [Waist-Hip Ratio] explode all trees OR MeSH descriptor: [Skinfold Thickness] explode all trees OR ("body mass" OR "body fat" OR "waist circumference" OR "hip circumference" OR "waist hip ratio" OR "waist to hip ratio" OR "skinfold thickness"):ti,ab,kw) ) ) NOT (MeSH descriptor: [Animals] explode all trees NOT MeSH descriptor: [Humans] explode all trees) ) NOT ((MeSH descriptor: [Infant] explode all trees OR MeSH descriptor: [Child] explode all trees OR MeSH descriptor: [Adolescent] explode all trees) NOT MeSH descriptor: [Adult] explode all trees) ) | | |
| Embase | ( ( ( (((ear/ OR ("ear" OR "auricular" OR "auricule").mp.) AND (acupressure/ OR ("acupress*" OR "finger press*" OR "point press*" OR "pressure bean?" OR "point taping" OR "point sticking" OR "ear seed?" OR "point seed?" OR "seed press*" OR "seed embedding" OR "acupoint*" OR "Shiatsu" OR "Zhi Ya").mp.)) OR ("ear pressing" OR "auricular pressing" OR "auriculoacupressure" OR "auriculoacupoint*" OR "auricular point*").mp.) OR (auricular acupuncture/ OR ((ear/ OR ("ear" OR "auricular" OR "auricule").mp.) AND "acupunctur*".mp.) OR "auriculoacupuncture".mp.) OR ("auriculotherapy" OR "auricular therapy").mp.) ) AND ( (obesity/ OR ("obesity" OR "obese").mp.) OR (body weight/ OR weight loss/ OR weight gain/ OR ("weight" OR overweight*).mp.) OR (body mass/ OR body fat/ OR waist circumference/ OR waist hip ratio/ OR skinfold thickness/ OR ("body mass" OR "body fat" OR "waist circumference" OR "hip circumference" OR "waist hip ratio" OR "waist to hip ratio" OR "skinfold thickness").mp.) ) ) NOT (animal/ NOT human/) ) NOT (juvenile/ NOT adult/) | 111 | 31 |
| CINAHL | ( ( ( ( ( (MH "Ear+") OR ( "ear" OR "auricular" OR "auricule" ) ) AND ( (MH "Acupressure+") OR ( "acupress*" OR "finger press*" OR "point press*" OR "pressure bean#" OR "point taping" OR "point sticking" OR "ear seed#" OR "point seed#" OR "seed press*" OR "seed embedding" OR "acupoint*" OR "Shiatsu" OR "Zhi Ya" ) ) ) OR ( "ear pressing" OR "auricular pressing" OR "auriculoacupressure" OR "auriculoacupoint*" OR "auricular point*" ) ) OR ( (MH "Acupuncture, Ear") OR ( ( (MH "Ear+") OR ( "ear" OR "auricular" OR "auricule" ) ) AND "acupunctur*" ) OR "auriculoacupuncture" ) OR ( (MH "Auriculotherapy+") OR ( "auriculotherapy" OR "auricular therapy" ) ) ) AND ( ( (MH "Obesity+") OR ( "obesity" OR "obese" ) ) OR ( ( (MH "Body Weight+") OR (MH "Weight Loss+") OR (MH "Weight Gain+") ) OR ( "weight" OR "overweight*" ) ) OR ( ( (MH "Body Mass Index") OR (MH "Waist Circumference") OR (MH "Waist-Hip Ratio") OR (MH "Skinfold Thickness") ) OR ( "body mass" OR "body fat" OR "waist circumference" OR "hip circumference" OR "waist hip ratio" OR "waist to hip ratio" OR "skinfold thickness" ) ) ) ) ) NOT ( (MH "Animals+") NOT (MH "Human") ) ) NOT ( ( (MH "Infant+") OR (MH "Child+") OR (MH | 88 | 4 |

| | | | |
|---|---|---|---|
| | "Adolescence+") ) NOT MH Adult+ ) | | |
| WoS | ( ( ( ( TS=("ear" OR "auricular" OR "auricule") AND TS=("acupress*" OR "finger press*" OR "point press*" OR "pressure bean$" OR "point taping" OR "point sticking" OR "ear seed$" OR "point seed$" OR "seed press*" OR "seed embedding" OR "acupoint*" OR "Shiatsu" OR "Zhi Ya") ) OR TS=("ear pressing" OR "auricular pressing" OR "auriculoacupressure" OR "auriculoacupoint*" OR "auricular point*") ) OR ( ( TS=("ear" OR "auricular" OR "auricule") AND TS=("acupunctur*") ) OR TS=("auriculoacupuncture") ) OR TS=("auriculotherapy" OR "auricular therapy") ) AND ( TS=("obesity" OR "obese") OR TS=("weight" OR "overweight*") OR TS=("body mass" OR "body fat" OR "waist circumference" OR "hip circumference" OR "waist hip ratio" OR "waist to hip ratio" OR "skinfold thickness") ) ) | 159 | 23 |
| Scopus | ( ( ( ( TITLE-ABS-KEY ( "ear" OR "auricular" OR "auricule" ) AND TITLE-ABS-KEY ( "acupress*" OR "finger press*" OR "point press*" OR "pressure bean*" OR "point taping" OR "point sticking" OR "ear seed*" OR "point seed*" OR "seed press*" OR "seed embedding" OR "acupoint*" OR "Shiatsu" OR "Zhi Ya" ) ) OR TITLE-ABS-KEY ( "ear pressing" OR "auricular pressing" OR "auriculoacupressure" OR "auriculoacupoint*" OR "auricular point*" ) ) OR ( ( TITLE-ABS-KEY ( "ear" OR "auricular" OR "auricule" ) AND TITLE-ABS-KEY ( "acupunctur*" ) ) OR TITLE-ABS-KEY ( "auriculoacupuncture" ) ) OR TITLE-ABS-KEY ( "auriculotherapy" OR "auricular therapy" ) ) AND ( TITLE-ABS-KEY ( "obesity" OR "obese" ) OR TITLE-ABS-KEY ( "weight" OR "overweight*" ) OR TITLE-ABS-KEY ( "body mass" OR "body fat" OR "waist circumference" OR "hip circumference" OR "waist hip ratio" OR "waist to hip ratio" OR "skinfold thickness" ) ) ) | 234 | 54 |
| Total number of retrieved articles | | 876 | 160 |
| Total number of retrieved articles | | 1036 | |

Supplementary S2.

**Table S2.** Full-text articles excluded after eligibility assessment and reasons for exclusion

| No | Reference | Reason for exclusion |
|---|---|---|
| 1 | E1 | Inappropriate research design |
| 2 | E2 | Weight loss is not dependent variable |
| 3 | E3 | Inappropriate research design |
| 4 | E4 | Inappropriate research design |
| 5 | E5 | Inappropriate research design |
| 6 | E6 | Inappropriate research design |
| 7 | E7 | Unsuitable target population |
| 8 | E8 | Inappropriate research design |
| 9 | E9 | Unavailable full articles |
| 10 | E10 | Other |
| 11 | E11 | Inappropriate research design |
| 12 | E12 | Inappropriate research design |
| 13 | E13 | Single group intervention |
| 14 | E14 | Unsuitable intervention |
| 15 | E15 | Inappropriate research design |
| 16 | E16 | Unsuitable intervention |
| 17 | E17 | Weight loss is not dependent variable |
| 18 | E18 | Single group intervention |
| 19 | E19 | Non-english or Korean articles |
| 20 | E20 | Other |
| 21 | E21 | Unsuitable target population |
| 22 | E22 | Unsuitable intervention |
| 23 | E23 | Inappropriate research design |
| 24 | E24 | Inappropriate research design |
| 25 | E25 | Inappropriate research design |
| 26 | E26 | Inappropriate research design |
| 27 | E27 | Inappropriate research design |
| 28 | E28 | Inappropriate research design |
| 29 | E29 | Inappropriate research design |
| 30 | E30 | Inappropriate research design |
| 31 | E31 | Inappropriate research design |
| 32 | E32 | Inappropriate research design |
| 33 | E33 | Inappropriate research design |
| 34 | E34 | Unsuitable target population |
| 35 | E35 | Inappropriate research design |
| 36 | E36 | Inappropriate research design |

| | | |
|---|---|---|
| 37 | E37 | Unsuitable intervention |
| 38 | E38 | Unavailable full articles |
| 39 | E39 | Inappropriate research design |
| 40 | E40 | Inappropriate research design |
| 41 | E41 | Single group intervention |
| 42 | E42 | Unsuitable target population |
| 43 | E43 | Non-english or Korean articles |
| 44 | E44 | Unavailable full articles |
| 45 | E45 | Unsuitable intervention |
| 46 | E46 | Unsuitable intervention |
| 47 | E47 | Unsuitable intervention |
| 48 | E48 | Unsuitable intervention |
| 49 | E49 | Single group intervention |
| 50 | E50 | Inappropriate research design |
| 51 | E51 | Unsuitable target population |
| 52 | E52 | Weight loss has not measured |
| 53 | E53 | Weight loss is not dependent variable |
| 54 | E54 | Non-english or Korean articles |
| 55 | E55 | Weight loss has not measured |
| 56 | E56 | Weight loss has not measured |
| 57 | E57 | Weight loss has not measured |
| 58 | E58 | Weight loss has not measured |
| 59 | E59 | Inappropriate research design |
| 60 | E60 | Unsuitable target population |
| 61 | E61 | Weight loss has not measured |
| 62 | E62 | Weight loss has not measured |
| 63 | E63 | Unsuitable intervention |
| 64 | E64 | Weight loss has not measured |
| 65 | E65 | Weight loss has not measured |
| 66 | E66 | Weight loss has not measured |
| 67 | E67 | Weight loss has not measured |
| 68 | E68 | Weight loss has not measured |
| 69 | E69 | Weight loss has not measured |
| 70 | E70 | Unsuitable intervention |
| 71 | E71 | Weight loss has not measured |
| 72 | E72 | Weight loss has not measured |
| 73 | E73 | Weight loss has not measured |
| 74 | E74 | Weight loss has not measured |
| 75 | E75 | Unsuitable target population |

| | | |
|---|---|---|
| 76 | E76 | Weight loss has not measured |

Note. E = Excluded study.

**Reference for Excluded Studies After Full-Text Assessment**


E1. Rahsepar, A.A.; Tavallaie, S.; Abdi, H.; Zhao, B.; Abbasi, P.; Nemati, M.; Safarian, M.; Parizadeh, S.M.R.; Mohammadi, M.; Darbandi, S.; et al. Effects of body acupuncture versus auricular acupuncture on anthropometric, lipid profile, inflammatory and immunologic markers: A randomized controlled trial study. *Clin. Biochem.* **2011**, 44, S156. https://doi.org/10.1016/j.clinbiochem.2011.08.388

E2. Bradford, A.; Tuffrey, V.; Polley, M. Effect of auricular acupuncture on appetite: A pilot study. Med. *Acupunct.* **2010**, 22, 181–186. https://doi.org/10.1089/acu.2010.0751

E3. Abdi, H. Effect of ear acupuncture on reducing weight and biochemical markers. *Clin. Biochem*. **2011**, 44, S235. https://doi.org/10.1016/j.clinbiochem.2011.08.573

E4. Hsieh, C.H. Auricular acupressure for weight reduction in obese Asian young adults: A randomized controlled trial. *Med. Acupunct.* **2007**, 19, 181–184. https://doi.org/10.1089/acu.2007.0542

E5. Hsieh, C.H.; Su, T.J.; Fang, Y.W.; Chou, P.H. Effects of auricular acupressure on weight reduction and abdominal obesity in Asian young adults: A randomized controlled trial. *Am. J. Chin. Med.* **2011**, 39, 433–440. https://doi.org/10.1142/S0192415X11008932

E6. Hsieh, C.H.; Su, T.J.; Fang, Y.W.; Chou, P.H. Efficacy of two different materials used in auricular acupressure on weight reduction and abdominal obesity. *Am. J. Chin. Med.* **2012**, 40, 713–720. https://doi.org/10.1142/S0192415X1250053X

E7. Hsu, C.H.; Wang, C.J.; Hwang, K.C.; Lee, T.Y.; Chou, P.; Chang, H.H. The effect of auricular acupuncture in obese women: A randomized controlled trial. *J. Womens Health* **2009**, 18, 813–818. https://doi.org/10.1089/jwh.2008.1005

E8. World Health Organization. ChiCTR2200056792: Acupoint catgut embedding under musculoskeletal ultrasonography combined with auricular point pressing with bean for weight control of middle-aged and elderly patients with abdominal obesity with metabolic syndrome. Available online: https://trialsearch.who.int/Trial2.aspx?TrialID=ChiCTR2200056792 (accessed on 21 March 2024).

E9. Abdi, H.; Ghayour, M. Effects of ear acupuncture on weight loss and serum levels of total cholesterol and triglyceride. *Avicenna J. Phytomed*. **2015**, 5, 1.

E10. Alp, H. Evaluation of anthropometric results after acupuncture and diet applications in obesity patients: An experimental-control study. *Acupunct. Electro-Ther. Res.* **2021**, 45, 97–106. https://doi.org/10.3727/036012921X16112663844905

E11. Abdi, H.; Ghaffarian-Zirak, R.; Barati, E.; Ghazizadeh, H.; Rohban, M.; Ghayour-Mobarhan, M. Effect of body and ear acupuncture on obesity. *Obes. Med.* **2020**, 19, 100257. https://doi.org/10.1016/j.obmed.2020.100257

E12. Fukui, J.A.; Rothwell, A.; Danesh, H.; Adelson, K.B.; Morris, G.J.; Irie, H.; Tiersten, A. Comparison of weight loss among early stage breast cancer patients post treatment: Nutrition education in combination with weight loss acupuncture vs. nutrition education alone. *J. Clin. Oncol.* **2018**, 36, e12594. https://doi.org/10.1200/JCO.2018.36.15_suppl.e12594

E13. Wang, J.; Li, Y.; Zhang, X.; Wang, M.N.; Cao, L.; Nu, N.; Li, Y.; Li, G.L. Ninety cases of simple obesity treated with the combined therapy of penetration needling, flash-fire cupping method and auricular acupuncture. *World J. Acupunct.-Moxibustion* **2018**, 28, 287–290. https://doi.org/10.1016/j.wjam.2018.12.005

E14. Lin, J.X.; Huang, Y.F.; Yin, M.Y.; Xu, M.Y.; Yin, L.; Lu, L.P.; Huang, D.M. Sixty cases of non-alcoholic fatty liver treated with TCM community comprehensive intervention. *Henan Tradit. Chin. Med.* **2015**, 35, 1830–1832.

E15. World Health Organization. KCT0008149: The effects of Auricular Acupressure on obesity and sleep in Korean Middle-aged women. Available online: https://cris.nih.go.kr/cris/search/detailSearchEn.do?seq=23867 (accessed on 21 March 2024).

E16. Rerksuppaphol, L. Efficacy of auricular acupressure combined with transcutaneous electrical acupoint stimulation for weight reduction in obese women. *J. Med. Assoc. Thai.* **2012**, 95, S32–S39.

E17. Darbandi, M.; Darbandi, S.; Owji, A.A.; Zhao, B.; Ghayour-Mobarhan, M.; Abdi, H.; Saberfiroozi, M.; Nematy, M.; Safarian, M.; Parizadeh, M.R.; et al. Auricular or body acupuncture: Which one is more effective in reducing abdominal fat mass in Iranian men with obesity: A randomized clinical trial. *J. Diabetes Metab. Disord.* **2014**, 13, 92. https://doi.org/10.1186/s40200-014-0092-3

E18. Chao, M.L.; Hsieh, C. The effect of an ear acupuncture program on the body mass index in obese females in Taiwan. *Thai J. Nurs. Res.* **2005**, 9, 103–110.

E19. Çelebi, M.M.; Akkurt, S.; Gençbay, M.B.; et al. The efficacy of auricular acupuncture application in the treatment of obesity. *Turk. J. Sports Med.* **2019**, 54, 45–51. https://doi.org/10.5152/tjsm.2019.115

E20. Cabıoğlu, M.T.; Ergene, N. Electroacupuncture therapy for weight loss reduces serum total cholesterol, triglycerides, and LDL cholesterol levels in obese women. *Am. J. Chin. Med.* **2005**, 33, 525–533. https://doi.org/10.1142/S0192415X05003132

E21. Rezvanimagham, M.; Faal Siahkal, S.; Ebrahimi, E. The effect of auriculotherapy on an overweight pregnant women's weight-gaining pattern: A randomized controlled clinical trial. *Evid.-Based Complement. Altern. Med.* **2023**, 2023, 7192142. https://doi.org/10.1155/2023/7192142

E22. Cabıoğlu, M.T.; Ergene, N.; Tan, U. Electroacupuncture treatment of obesity with psychological symptoms. *Int. J. Neurosci.* **2007**, 117, 579–590. https://doi.org/10.1080/00207450500535545

E23. ClinicalTrials.gov. NCT00414505: Evaluation of Carisbamate for the Treatment of Migraine in a Randomized, Double-Blind Trial. Available online: https://clinicaltrials.gov/study/NCT00414505 (accessed on 21 March 2024).

E24. ClinicalTrials.gov. NCT02326714: A Phase II Study of Pembrolizumab (MK-3475) in Participants With Advanced/Metastatic Urothelial Carcinoma. Available online: https://clinicaltrials.gov/study/NCT02326714 (accessed on 21 March 2024).

E25. ClinicalTrials.gov. NCT02269371: Study of Pembrolizumab (MK-3475) Versus Chemotherapy or Pembrolizumab Plus Chemotherapy in Advanced Gastric or Gastroesophageal Junction Adenocarcinoma (KEYNOTE-062). Available online: https://clinicaltrials.gov/study/NCT02269371 (accessed on 21 March 2024).

E26. ClinicalTrials.gov. NCT02438540: Electroacupuncture for Postoperative Ileus After Laparoscopic Resection of Colorectal Cancer. Available online: https://clinicaltrials.gov/study/NCT02438540 (accessed on 21 March 2024).

E27. ClinicalTrials.gov. NCT04089384: Efficacy and Safety of Acupuncture/Acupressure Clinical Study. Available online: https://clinicaltrials.gov/study/NCT04089384 (accessed on 21 March 2024).

E28. ClinicalTrials.gov. NCT02840916: Clinical Trial of Acupuncture Evaluation. Available online: https://clinicaltrials.gov/study/NCT02840916 (accessed on 21 March 2024).

E29. ClinicalTrials.gov. NCT00969267: Clinical Study Registration for Treatment Protocol. Available online: https://clinicaltrials.gov/study/NCT00969267 (accessed on 21 March 2024).

E30. ClinicalTrials.gov. NCT01102075: Clinical Evaluation Trial Protocol. Available online: https://clinicaltrials.gov/study/NCT01102075 (accessed on 21 March 2024).

E31. ClinicalTrials.gov. NCT01167348: Clinical Trial Registration. Available online: https://clinicaltrials.gov/study/NCT01167348 (accessed on 21 March 2024).

E32. ClinicalTrials.gov. NCT06091761: Clinical Trial Study Registration. Available online: https://clinicaltrials.gov/study/NCT06091761 (accessed on 21 March 2024).

E33. Wei, Q.L.; Liu, Z.C. Treatment of simple obesity with auricular acupuncture, body acupuncture and combination of auricular and body acupuncture. *Chin. J. Clin. Rehabil.* **2004**, 8, 4357–4359.

E34. Wei, Q.L. Comparison of the therapeutic effect of auricular acupuncture, body acupuncture and combination of auricular plus body acupuncture for treatment of simple obesity. *World J. Acupunct.-Moxibustion* **2001**, 11, 28.

E35. Giller, R.M. Auricular acupuncture and weight reduction. A controlled study. *Am. J. Acupunct.* **1975**, 3, 151–153.

E36. Darbandi, S.; Darbandi, M.; Ghayour Mobarhan, M.; Mokaram, P.; Owji, A.A.; Saberfiroozi, M.; Abdi, H.; Nematy, M.; Safarian, M.; Parizadeh, M.R.; et al. The effects of auricular acupuncture on leptin hormone in Iranian obese and overweight subjects. *Clin. Biochem.* **2011**, 44, S128. https://doi.org/10.1016/j.clinbiochem.2011.08.302

E37. Darbandi, S.; Darbandi, M.; Mokarram, P.; Owji, A.A.; Zhao, B.; Ghayor-Mobarhan, M.; Abdi, H.;

Saberfiroozi, M.; Nematy, M.; Safarian, M.; et al. Effects of body electroacupuncture on plasma leptin concentrations in obese and overweight people in Iran: A randomized controlled trial. *Altern. Ther. Health Med.* **2013**, 19, 24–31.

E38. Wang, S.; Liang, R.; Xie, P.; Zhang, S.; To, P. A clinically controlled study on the effect of acupuncture and auricular-point tapping and pressing therapy for the treatment of obesity. *Int. J. Clin. Acupunct*. **2007**, 16, 231–237.

E39. Fujimoto, T.; Masuda, H.; Hataoka, T.; Kobayashi, H. Determination of slimming effect by auricular acupuncture stimulation-statistical analysis of 1,017 female data. Vnitr. Lek. **2017**.

E40. Koskenniemi, T. Effect of ear acupuncture for obesity. *Acupunct. Electro-Ther. Res*. **1985**, 10, 231.

E41. Shiraishi, T.; Onoe, M.; Kojima, T.A.; Kageyama, T.; Sawatsugawa, S.; Sakurai, K.; Yoshimatsu, H.; Sakata, T. Effects of bilateral auricular acupuncture stimulation on body weight in healthy volunteers and mildly obese patients. *Exp. Biol. Med.* **2003**, 228, 1201–1207. https://doi.org/10.1177/153537020322801016

E42. Qunli, W.; Zhicheng, L. Acupuncture treatment of simple obesity. *J. Tradit. Chin. Med*. **2005**, 25, 90–94.

E43. Qiu, X.L.; Li, D.Q. Analysis on adipose improvement after acupuncture treating simple obesity. *World Chin. Med.* **2014**, 9, 1352–1354.

E44. Tang, X. 75 cases of simple obesity treated with auricular and body acupuncture. *J. Tradit. Chin. Med.* **1997**, 17, 55–56.

E45. Bei, Y.; Fang, X.; Yao, Z. Sixty-two cases of simple obesity treated by acupuncture combined with massage. *J. Tradit. Chin. Med*. **2004**, 24, 36–39.

E46. Beyazit, Y.; Kekilli, M.; Purnak, T.; Spiegel, J. Ear stapling application as an alternative treatment for weight loss. *Ear Nose Throat J.* **2011**, 90, 294–296. https://doi.org/10.1177/014556131109000702

E47. Hung, Y.C.; Hung, I.L.; Hu, W.L.; Tseng, Y.J.; Kuo, C.E.; Liao, Y.N.; Wu, B.Y.; Chen, H.P.; Huang, M.H.; Su, F.Y.; et al. Reduction in postpartum weight with laser acupuncture: A randomized control trial. *Medicine* **2016**, 95, e4716. https://doi.org/10.1097/MD.0000000000004716

E48. Soong, Y.S. The treatment of exogenous obesity employing auricular acupuncture. *Am. J. Chin. Med*. **1975**, 3, 285–287. https://doi.org/10.1142/S0192415X75000311

E49. Zhang, Z. Weight reduction by auriculo-acupuncture--a report of 110 cases. *J. Tradit. Chin. Med*. **1990**, 10, 17–18.

E50. Ear acupuncture helps reduce waist circumference. *J. Chin. Med*. **2020**, 122, 67.

E51. Hsieh, C.H. The effects of auricular acupressure on weight loss and serum lipid levels in overweight adolescents. *Am. J. Chin. Med*. **2010**, 38, 675–682. https://doi.org/10.1142/S0192415X10008147

E52. Adel, H.E.; Abbassy, A.H.; Yousef, A.M. Effect of reducing abdominal obesity on low back pain in post natal women. *Turk. J. Physiother. Rehabil*. **2021**, 32, 3.

E53. Ito, H.; Yamada, O.; Kira, Y.; Tanaka, T.; Matsuoka, R. The effects of auricular acupuncture on weight reduction and feeding-related cytokines: A pilot study. *BMJ Open Gastroenterol.* **2015**, 2, e000013. https://doi.org/10.1136/bmjgast-2014-000013

E54. Binesh, M.; Paivastegar, M.; Hashem Dabaghian, F.; Dehshiri, G. Comparison of the effectiveness of mindfulness intervention, auricular acupuncture, and their combined intervention on eating behavior styles and body mass index in women with obesity: A clinical study. Iran. *J. Health Educ. Health Promot*. **2025**, 13, 46–63.

E55. Garramona, F.T.; Moreira, R.S.O.; Korch, H.; Cruz, J.E.; Teixeira, L.F.M. Enhancing flexibility through Chinese auriculotherapy: Investigating the impact on sit and reach test–a randomized controlled trial. *Tradit. Integr. Med*. **2024.**

E56. Fathima, L.; Prabu, D.; Rajmohan, M. Evaluating the quality and impact of nicotine replacement therapy, auricular acupressure, and structured health education and training given by dentist for smoking cessation: A prospective interventional study among smokers in Chennai, Tamil Nadu, India. *Glob. J. Med. Pharm. Biomed. Update* **2025**, 20, 30–31. https://doi.org/10.25259/GJMPBU_32_2025_075

E57. Wang, Y.; Rong, Z.; Wang, P.; Meng, J.; Yang, F.; Xie, R.; Deng, Z.; Yuan, S.; Guo, T. Auricular acupressure combined with Tongtian oral liquid for acute attacks of migraine without aura: A single-center, retrospective study. *Am. J. Transl. Res*. **2024**, 16, 625–636.

E58. Han, Z.; Tang, H.; Hou, M.; Gao, L.; Dong, Z.; Wang, C.; Lin, Q.; Yang, W. Auricular acupressure in relieving PONV and promoting gastrointestinal function recovery in females after laparoscopic sleeve gastrectomy: A prospective randomized controlled trial. *Obes. Surg.* **2024**, 34, 1–10. https://doi.org/10.1007/s11695-024-07243-z

E59. Koda, E.K. Electrical stimulation of acupuncture points helps resolve a child's failure to thrive. *Med. Acupunct*. **2024**, 36, 102–107. https://doi.org/10.1089/acu.2023.0087

E60. Zhang, Y.; Sun, S.; Zhao, D.; Yang, J.; Wang, S. A novel intelligent physiotherapy robot based on dynamic acupoint recognition method. *Front. Neurorobot.* **2025**, 19, 1696824. https://doi.org/10.3389/fnbot.2025.1696824

E61. Urlesberger, B.; Cabano, R.; Soll, G.; Pahl, A.; Oei, J.L.; Schmölzer, G.M.; Raith, W.; Bruschettini, M. Acupuncture for neonatal abstinence syndrome in newborn infants. *Cochrane Database Syst. Rev*. **2025**, 2, CD014160. https://doi.org/10.1002/14651858.CD014160.pub2

E62. Liu, K.J.; Chen, Y.; Yang, W.; Peng, X.M. Buccal acupuncture: A potential treatment for postoperative nausea and vomiting in patients undergoing metabolic and bariatric surgery. *Obes. Surg.* **2024**, 34, 4275–4276. https://doi.org/10.1007/s11695-024-07513-w

E63. Jang, S.; Ko, M.M.; Kim, B.Y.; Son, M.J.; Jegal, K.H.; Park, J.H.; Kim, S. Development of an adverse event reporting template for Ephedrae Herba for weight loss. *Eur. J. Integr. Med*. **2024**, 67, 102341. https://doi.org/10.1016/j.eujim.2024.102341

E64. Khodabakhshian, F.; Tagharrobi, Z.; Sharifi, K.; Sooki, Z.; Momen-Heravi, M.; Joshaghani, F.Z. Effect of auriculotherapy on persistent fatigue in recovered patients from the acute phase of COVID-19: A double-blind randomized clinical trial. *Fatigue Biomed. Health Behav*. **2025**, 13, 54–66. https://doi.org/10.1080/21641846.2024.2395230

E65. Zhang, Y.; Wu, S.; Zhu, K.; Li, K.; Qi, Z.; Zhu, B.; Hou, S.; Wang, W. Effects of auriculotherapy on urinary incontinence in men with prostate cancer: A randomized controlled trial. *BMC Complement. Med. Ther.* **2025**, 25, 4979. https://doi.org/10.1186/s12906-025-04979-8

E66. Sagui, E.; Claverie, D.; Bidaut, W.; Grelot, L. Heart rate variability and cold-induced vascular dilation after stimulation of two different areas of the ear: A prospective, single-blinded, randomized crossover study. *BMC Complement. Med. Ther.* **2024**, 24, 4392. https://doi.org/10.1186/s12906-024-04392-7

E67. Fu, C.; Hou, X.; Zheng, C.; Zhang, Y.; Gao, Z.; Yan, Z.; Ye, Y.; Liu, B. Immediate modulatory effects of transcutaneous vagus nerve stimulation on patients with Parkinson's disease: A crossover self-controlled fMRI study. *Front. Aging Neurosci.* **2025**, 17, 1444703. https://doi.org/10.3389/fnagi.2024.1444703

E68. Kambam, K.K.; Kumar, R.; Kumar, S.; Mahesh, M.; Reddy, V.K.; et al. Magnetic non-invasive auricular acupuncture during eye-exam for retinopathy of prematurity in preterm infants: A multicentre randomized controlled trial. *TPM-Test. Psychom. Methodol. Appl. Psychol.* **2025**, 32 (S4), 1348–1361.

E69. Hamilton, K.R.; Lukkahatai, N.; Chen, W.; Wu, H.; Kawi, J.; Johnson, C.M.; Christo, P.J.; Campbell, C.M. Pain phenotyping indicators in older adults with chronic low back pain: A secondary analysis of a randomized controlled trial. *J. Pain Res.* **2025**, 18, 4589–4598.

E70. Lemos, J.F.; Araújo, L.M.C.; Guimarães-do-Carmo, V.J.; Cardoso, E.J.A.; da Silva Ferreira, A.I.; Barbosa, K.F.D.S.; Melo, R.S. Sedentary behavior, increasing age, and overweight/obesity increase the presence and intensity of the chronic joint pain in individuals affected by Chikungunya fever. *Clin. Rheumatol.* **2024**, 43, 2993–3003.

E71. Pham, T.T.L.; Le, N.T.; Nguyen, T.T.; Chu, H. Should thread-embedding and auricular acupuncture be combined rather than used individually for non-specific chronic low back pain?: A double-blinded, randomized, sham-controlled trial. *Integr. Med. Res.* **2025**, 14, 100998.

E72. Ali, R.A.; Obeidat, R.F.; Oweis, A.I. The effect of auricular massage on naso-oral suctioning procedural pain in premature neonates: A randomized controlled crossover study. *ScientificWorldJournal* **2025**, 2025, 8819743. https://doi.org/10.1155/tswj/8819743

E73. Mattar, J.G.; Chalah, M.A.; Abi, L.G.N.; Ayache, S.S.; Sorel, M.; Lefaucheur, J.P.; Ouerchefani, N.; Le, G.J. The effect of the EXOPULSE Mollii Suit on pain and fibromyalgia-related symptoms—A randomized sham-controlled crossover trial. *Eur. J. Pain* **2025**, 29, e4729. https://doi.org/10.1002/ejp.4729

E74. Liu, Q.; Shi, L.; Lin, F.; Wang, Z.; Zhang, S.; Chen, L.; Zhan, M.; Zhang, H.; Miao, C. Understanding the effect of acupuncture on nausea and vomiting during pregnancy from a metabolic perspective: Study protocol for a single-blinded randomized controlled trial. *BMC Complement. Med. Ther*. **2024**, 24, 4656.

https://doi.org/10.1186/s12906-024-04656-2

E75. Sun, W.; He, J.; Wang, W.; Lu, C.; Lin, Y.; Yu, J.; Dou, Y.; Yan, W. Auricular acupressure versus an intermittent low-carbohydrate diet in children with overweight or obesity with gastric-heat and dampness-obstruction syndrome: Protocol for a randomized controlled trial. *JMIR Res. Protoc.* **2025**, 14, e59856. https://doi.org/10.2196/59856

E76. de la Barra Ortiz, H.A.; Chamorro Lange, C.; Liebano, R.E. Effectiveness of low-level laser auriculotherapy in the treatment of myogenic temporomandibular disorders and anxiety: A randomized, placebo-controlled trial. *Int. J. Environ. Res. Public Health* **2026**, 23, 697. https://doi.org/10.3390/ijerph23060697
